# Dysregulated tumor-associated macrophages in carcinogenesis, progression and targeted therapy of gynecological and breast cancers

**DOI:** 10.1186/s13045-021-01198-9

**Published:** 2021-10-30

**Authors:** Tianhan Xu, Sihui Yu, Jiawen Zhang, Sufang Wu

**Affiliations:** 1 Department of Obstetrics and Gynecology, Shanghai General Hospital, Shanghai Jiao Tong University School of Medicine, Shanghai, People’s Republic of China; 2 Reproductive Medicine Center, Department of Obstetrics and Gynecology, Shanghai General Hospital, Shanghai Jiao Tong University School of Medicine, Shanghai, People’s Republic of China

**Keywords:** Tumor microenvironment, Tumor-associated macrophage, Gynecological cancers, Breast cancer, Targeted therapy

## Abstract

Gynecological and breast cancers are a group of heterogeneous malignant tumors. Although existing treatment strategies have ameliorated the clinical outcomes of patients, the overall survival rate of advanced diseases remains unsatisfactory. Increasing evidence has indicated that the development and prognosis of tumors are closely related to the tumor microenvironment (TME), which restricts the immune response and provokes malignant progression. Tumor-associated macrophages (TAMs) are the main component of TME and act as a key regulator in tumor metastasis, immunosuppression and therapeutic resistance. Several preclinical trials have studied potential drugs that target TAMs to achieve potent anticancer therapy. This review focuses on the various functions of TAMs and how they influence the carcinogenesis of gynecological and breast cancers through regulating cancer cell proliferation, tumor angiogenesis and tumor-related immunosuppression. Besides, we also discuss the potential application of disabling TAMs signaling as a part of cancer therapeutic strategies, as well as CAR macrophages, TAMs-based vaccines and TAMs nanobiotechnology. These research advances support that targeting TAMs combined with conventional therapy might be used as effective therapeutics for gynecological and breast cancers in the future.

## Introduction

According to the global cancer statistics in 2020, the total number of gynecological tumors, including cervical, vulvar, corpus uteri, ovarian and vaginal cancers, accounts for up to 15% overall new morbidity in women worldwide. Additionally, breast cancer also accounts for about a quarter of all new cancer cases and one in six cancer death cases in women [1]. Although combined surgery with or without chemoradiotherapy, immunotherapy and targeted therapy have greatly ameliorated the clinical outcomes of patients with gynecological and breast cancers, the overall survival rate of advanced diseases is still low.

In recent years, a new concept and field about tumor microenvironment (TME) has become a hot topic in cancer researches. Tumor cells are surrounding by a dynamic environment which includes macrophages, lymphocytes, mesenchymal stem cells, fibroblasts as well as other immune, inflammatory and stromal cells and a variety of biochemical molecules. Among these components of TME, the tumor-associated macrophages (TAMs) are the main actors, accounting for 30% to 50% of TME cells [2], which play a crucial role in angiogenesis, tumor metastasis, immunosuppression and therapeutic resistance [3]. Interestingly, gynecological tumors have different cross talk with TAMs due to their characteristics. For example, persistent infection with high-risk human papillomavirus (HPV) is one of the main causes of cervical cancer. HPV E6/E7 oncoproteins not only change cell proliferation and interferon response by targeting cytokine expression [4, 5], but also have multiple associations with TAMs together in furtherance of tumor development [2]. Besides, estrogen receptors, which are highly expressed in endometrial, ovarian and breast cancers, interact with TAMs, contributing to migration and invasion of cancer cells [6, 7]. In our previous studies, we also found the correlation between TAMs infiltration and gynecological cancers [8–10].

Therefore, in this review, we will focus on the TAMs in gynecological and breast cancers with the emphasis on their origin, function, and regulation of tumor progression and metastasis. We also review the promising TAMs target therapies to inhibit pro-tumor TAMs, and novel TAMs-based immunotherapies including CAR macrophages, vaccination and nanobiotechnology which are in full flourish.

### Origin and development of TAMs

**Fig. 1:**
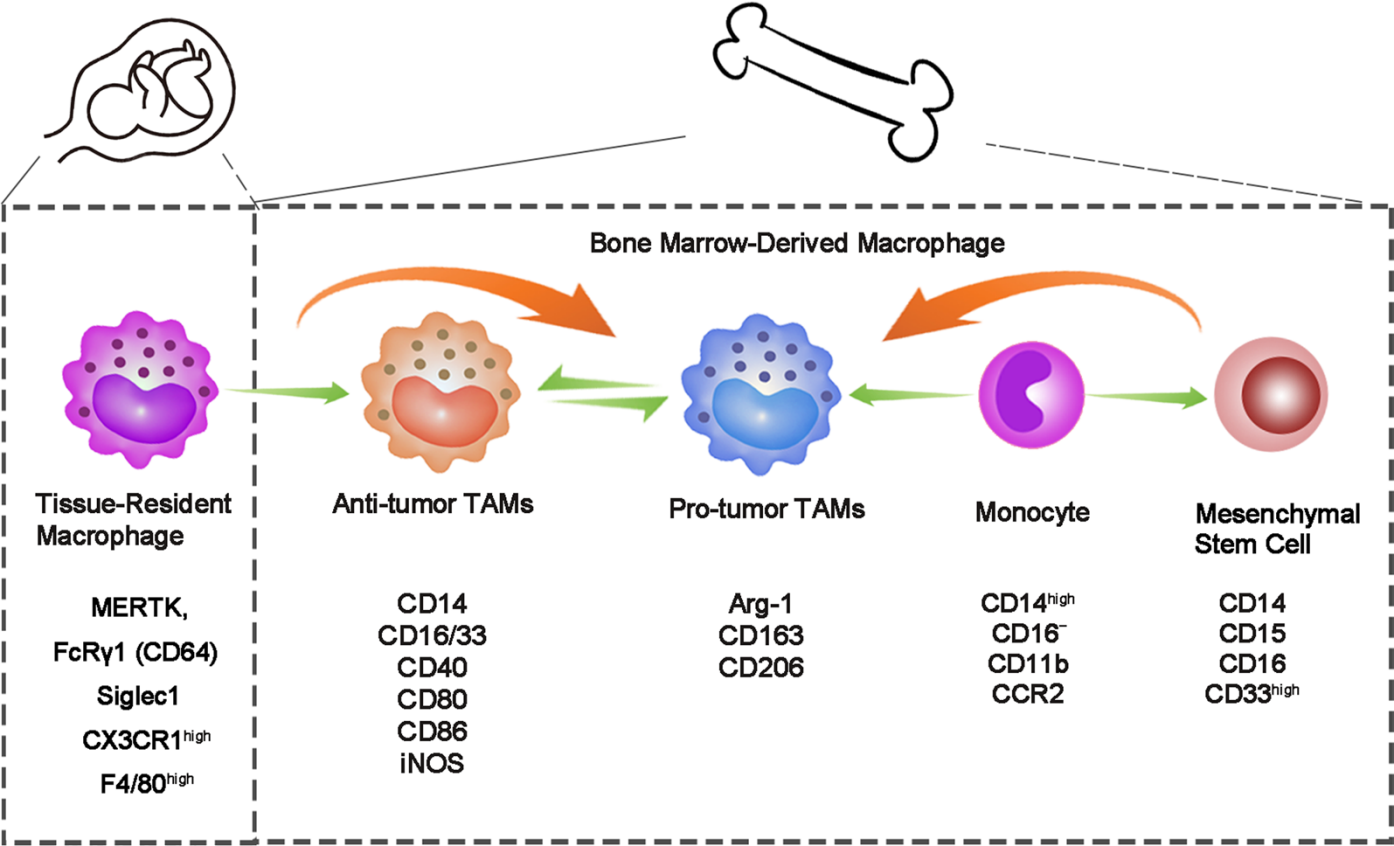
Surface markers of TRMs and BMDMs

As a vital component of innate immune system, macrophages belong to mononuclear phagocytic system which can engulf pathogens, inhibit inflammation and coordinate tissue repair [11, 12]. The macrophages in tissues are composed of two parts: yolk sac-derived tissue-resident macrophages (TRMs) and blood monocytes from bone marrow [13]. TAMs are the macrophages gathered in TME [14], and the majority of TAMs are bone marrow-derived macrophages (BMDMs) recruited by chemotactic and cytokines such as monocyte chemotactic protein 1 (MCP-1/CCL2) and colony-stimulating factor 1 (CSF-1) [3]. In general, hematopoietic stem cells (HSC) differentiate into bone marrow progenitor cells (CMP), then go through granulocyte/macrophage-restricted progenitor cells (GMP) and macrophage/dendritic progenitors (MDP), and have the ability to differentiate into monocytes and mononuclear myeloid suppressor cells (M-MDSC) [15]. Once these cells enter the TME, local inducers gradually regulate the differentiation of monocytes into pro-tumor- or anti-tumor-type macrophages (Table 1) [16–31]. TAMs with different origin can be distinguished by the expression of surface markers (such as CX3CR1, CD45, CCR2, MERTK and FcRγ1/CD64), gene expression profile and lineage trackers (Fig. 1) [13].  Besides, different functions of TAMs have been found, for example, TRMs are related to cell proliferation and fibrosis, while BMDMs are related to antigen presentation [32], which affects the growth and metastasis of tumor cells to some extent and provides research directions for possible cancer therapeutic strategies [33, 35, 36].

**Table 1:** TAMs differentiation under the influence of tumor microenvironment

Tumor type	Pathway	Differentiation	References
Breast cancer	Lactate/Gpr132	Pro-M2 polarization	[16]
	Lactate/ERK/STAT3	Pro-M2 polarization	[17]
	Emodin/STAT6/C/EBPβ	Anti-M2 polarization	[18]
	MCT-1/miR-34a/IL-6/IL-6R	Pro-M2 polarization	[19]
	miR-200c/PAI-2	Pro-M2 polarization	[20]
Ovarian cancer	CTHRC1/STAT6	Pro-M2 polarization	[21]
	PPARγ/NF-κB	Pro-M2 polarization	[22]
Endometrial cancer	miRNA-21	Pro-M2 polarization	[48]
	lncRNA NIFK-AS1	Anti-M2 polarization	[23]
	rCTHRC1/CX3CR1	Pro-M2 polarization	[24]
Cervical cancer	Interleukin-17/COX-2/PGE2	Pro-M2 polarization	[25]
Lung cancer	Succinate/SUCNR1/PI3K-HIF-1α	Pro-M2 polarization	[26]
Colorectal cancer	PKN2/DUSP6-Erk1/2	Anti-M2 polarization	[27]
	PKCα/MKK3/6-P38 MAPK	Pro-M1 polarization	[28]
	EGFR/PI3K/AKT/mTOR	Pro-M2 polarization	[29]
	SPON2/integrin β1/PYK2	Pro-M2 polarization	[30]
Gastric cancer	TLR4/PI3K/Akt	Pro-M2 polarization	[31]

### The polarization of TAMs

According to different polarization factors and immune functions, macrophages can be divided into two types, namely classically activated M1 and alternatively activated M2 macrophages [37]. M1 macrophages are mainly induced by interferon γ (IFNγ), lipopolysaccharide (LPS) and granulocyte-macrophage colony-stimulating factor (GM-CSF), and have cytotoxic effects on cancer cells. On the contrary, M2 macrophages can provide a nutritional advantage to cancer cells when stimulated by IL-4, IL-6, IL-10, IL-13, transforming growth factor β (TGF-β), vascular endothelial growth factor (VEGF) and other M2-specific cytokines [34, 38, 39]. It is generally assumed that TAMs are largely polarized into M2 macrophages, promoting tumor progression and restraining anti-tumor immune response.

This polarization trend can be attributed to various inducible factors secreted by constituent cells in TME (Table 1). For example, Pedraza-Brindis et al. reported that the supernatant of cervical cancer cells down-regulated the phosphorylation of NF-κB-p65 and STAT1 in M1 macrophages and induced a stable M2 phenotype, accompanied by increased expression of IL-10, IL-6, CCL2, IL-8, GM-CSF, G-CSF, platelet-derived growth factor (PDGF) AA and PDGF-BB [40, 41]. Experiments have shown that cervical cancer cells secreted prostaglandin (PG) E2 and IL-6 to induce M2 phenotype and produce CCL2 to promote the recruitment of monocytes at the tumor site [34]. Moreover, CSF-1 can induce M2 polarization in ovarian cancer cells with the progression of histological malignancy [42]. In addition, blocking CSF-1 receptor (CSF-1R) can prevent macrophage infiltration and endometrial cancer cell proliferation [43]. What’s more, paclitaxel treatment of breast cancer cells enhances the secretion of CSF-1, recruiting TAMs so as to limit the therapeutic effect [44].

### Effects of anoxic microenvironment on TAMs

Rapid proliferation of tumor cell results in insufficiency of blood flow, lactic acid accumulation and hypoxia in TME, which leads to more angiogenesis and catalyzes tumor growth [49]. In the TME with low oxygen concentration, tumor cells can recruit TAMs by secreting more chemokines such as CCL2, CCL5, or CSF1 [3]. In the TME of cervical cancer, the hypoxic condition was confirmed to activate M2-like TAMs in vivo [45]. One previous study revealed that macrophages were induced to hypoxic tumor areas and gained a M2-polarized phenotype via hypoxic cancer cell-derived cytokines Oncostatin M (OSM), an inflammatory cytokine belonging to the IL-6 superfamily. Recently, this phenomenon has been proven to be mediated by mTOR signaling complex 2 (mTORC2) [46, 47]. In addition, studies on exosomes have discovered that endometrial cancer with hypoxia mediated the polarization of monocyte into M2 macrophages through exuding exocrine miRNA-21 [48]. Meanwhile, hypoxic TME also leads to decreased extracellular matrix mobility and slower TAMs movement, which enhances the interaction between TAMs and cancer cells [50].

Experiments were also carried out to demonstrate whether hypoxia-induced ZEB1, a key regulator of epithelial–mesenchymal transformation (EMT), plays a part in TAMs recruitment in the hypoxic cervical cancer TME. The results showed that ZEB1 up-regulated CCL8 which combined with CCL8 receptor (CCR2) and further recruited TAMs though NF-κB signaling phosphorylation [51].

### TAM biomarkers

Cell surface markers are a class of substances, usually proteins, that are expressed on the surface of cells to identify cell types or properties. There are many kinds of receptors on the surface of macrophages, among which Tyro3, Axl and MerTK receptor tyrosine kinases were studied far and wide [52]. When these receptors are paired with ligands such as growth inhibition specificity 6 (Gas6) and S protein, macrophages are encouraged to polarize to M2 through the conduction of specific pathways, among which the PI3K/Akt signaling is the most studied pathway [53–55].

Except from receptors on macrophage, these cells also express MHC-II and CD on cytomembrane to participate in the immune response. In general, M1 macrophages express CD14, CD16/33, CD40, CD80 and CD86, produce IL-6, IL-12p70 and IL-23, and play pro-inflammatory, cytotoxic and tumoricidal roles. As for M2 macrophages, arginase 1 (Arg-1), CD163, CD169, CD206, IL-10 and chemokines CCL17 and CCL22 have been identified as markers [57, 58]. Pan-macrophage markers have also been involved in the detection of macrophage cells, such as CD68, CD14, CD45, CD105 and CD204 [14, 40]. Many years ago, some publications simply included TAMs in M2-like macrophage [56], which is no longer applicable today. TAMs tend to polarize to M2 in TME, and their surface receptors and cytokines secreted are similar to M2-like macrophages. Meanwhile, TAMs also express some M1-like macrophage markers. For example, iNOS (+) TAMs were detected to be more beneficial to the prognosis of cancer patients [59]. Besides, the effect of TAMs in stimulating T cell activity with high expression of M2-like markers in ovarian ascites was similar to that of M1-like TAMs [60]. Therefore, we cannot simply classify TAMs as M1- or M2-like macrophages and recognized their function based solely on the expression of surface markers.

In a word, TAMs biomarkers help to detect the presence and function of macrophages in tumor tissues (Table 2), which may facilitate the identification, diagnosis and treatment of diseases. The search and discovery of valuable TAMs biomarkers have become an important research hot spot.

**Table 2:** TAMs markers

TAM marker	Tumor type	Function	References
CD68	Breast cancer	The expression of CD68 was negatively correlated with ERα or progesterone receptor	[7]
CD14, CD163	Cervical cancer	CD14^+^CD163^+^macrophages have poor ability to stimulate T cell proliferation and produce INF-γ	[34]
CD163, CD204	Ovarian cancer	Tumor-derived CSF-1 may induce CD163^+^CD204^+^M2 differentiation	[42]
Arg-1, CD206, iNOS, CD86	Endometrial cancer	Endometrial cancer cells induced high expression of Arg-1 and CD206 and low expression of iNOS and CD86 on TAMs	[43]
CD163	Cervical cancer	Hypoxia is positively associated with CD163^+^TAM infiltration and cervical cancer progression	[45]
CD163, CD206	Breast cancer	mTORC2 mediated CD163^+^CD206^+^M2 polarization of macrophages in hypoxic TME	[47]
CD206	Endometrial cancer	Endometrial cancer cells promoted CD206^+^M2-like macrophage polarization	[48]

### The interaction of TAMs and other immune and stromal cells in TME

**Fig. 2:**
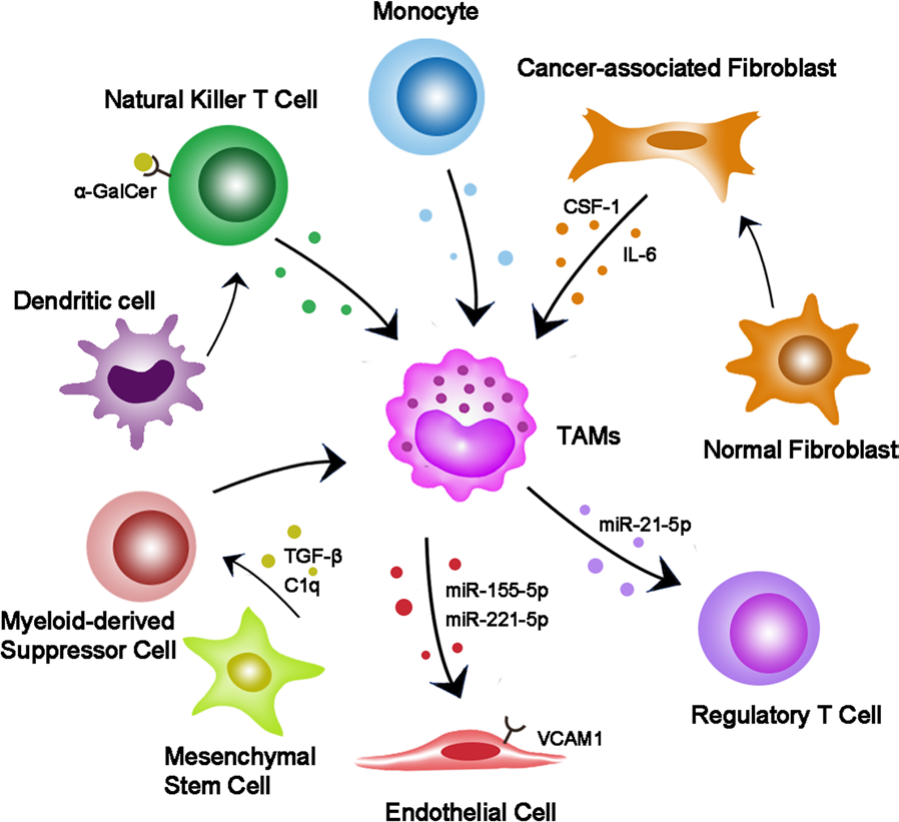
The underlying roles of TAMs in tumor. TAMs consist of M1 and M2 macrophages, and M2 is the dominant type induced by TME components. TAMs promote tumor cell proliferation, angiogenesis, metastasis and immunosuppression through cytokine secretion, exosome release and ECM remodeling. Concurrently, tumor cells can also recruit macrophages and reeducate TAMs

TAMs interact widely with other microenvironment cells in TME (Fig. 2). Cancer-associated fibroblasts (CAFs), which mainly originate from normal fibroblasts in the interstitial tissue around the tumor, are closely related to TAMs [61]. It was found that macrophages co-cultured with CAFs increased the ability to differentiate into M2-like macrophages via IL-6 and CSF-1 secreted by CAFs [62]. In the experiment of three-way cross talk among cancer cells, CAFs and monocytes, the group with cancer cell conditioned medium (CM) showed the highest level of M2-like differentiation [62]. At the same time, CAFs and TAMs activate each other, which play a synergistic role in promoting tumor cell invasion and angiogenesis [63].

For endothelial cells, the exosomes derived from macrophages can promote their proliferation and increase the vascular density of tumor [64]. On the other hand, studies have shown that M2-like macrophages in ovarian cancer directly contact with endothelial cells to enhance vascular barrier and reduce vascular permeability to restrict ascites formation [65].

For T cells, TAMs not only up-regulated the expression of PD-L1, but also decreased the proportion of activation and proliferation of CD8^+^T cells [66]. Some studies have shown T cell elimination in liver metastasis, which was mediated by macrophages [67]. Regulatory T (Treg) and T helper 17 (Th17) cells are two subsets of CD4^+^ T cells. The imbalance of Treg/Th17 cells is one of the characteristics of tumors [68]. In ovarian cancer, the exosomes from M2 macrophages which contain hsa-miR-21-5p increase the Treg/Th17 ratio and promote tumor progression and metastasis to the peritoneum [68]. On the other hand, Treg cells can preferentially promote M2-like TAMs by inhibiting the secretion of IFN-γ by CD8^+^ T cells [69]. In addition, activation of natural killer T cells increases iNOS^+^CD206^−^ M1 macrophages and controls the growth of solid tumors [70].

Myeloid-derived suppressor cells (MDSCs) are immature bone marrow cells with the morphological and phenotypic characteristics of neutrophils and monocytes [71]. Blocking TAMs can contribute compensatory infiltration of G-MDSC, thus counteracting the reduction in tumor load by inhibiting TAMs [72]. Additionally, exosomes released by tumor-induced mesenchymal stem cells (MSC) accelerated the progression of breast cancer by inducing M-MDSC to differentiate into M2 macrophages in TME [73]. Moreover, evidence also suggests that tumor exosomes can differentiate MDSC into M2 macrophages as well [74].

### The relationship of TAMs with gynecological and breast cancers

**Fig. 3:**
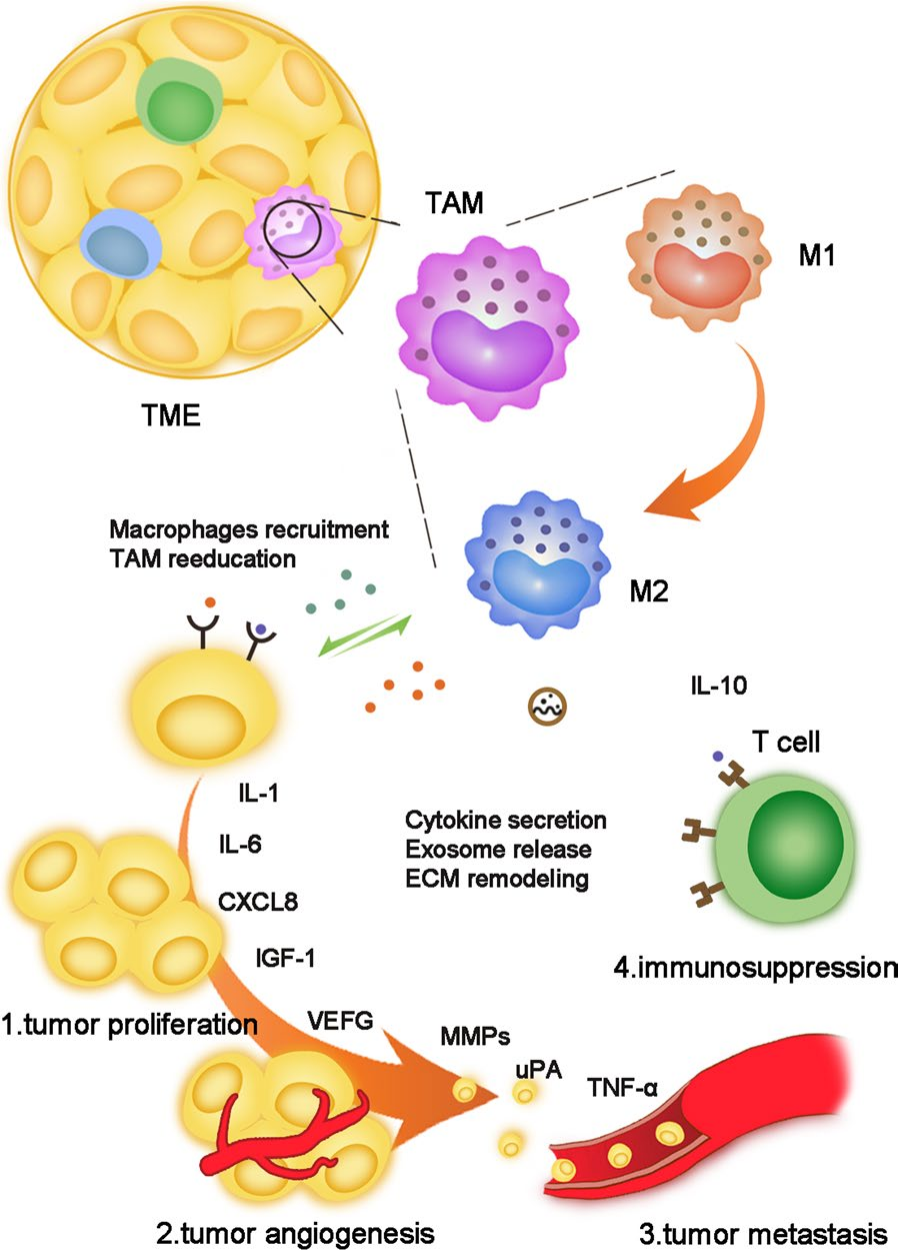
The interaction of TAMs and other immune and stromal cells in TME

The emerging new evidences suggest that TAMs participate in different stages and aspects of tumor development by secreting diverse factors. These molecules, such as IL-1/6/8/10, IGF-I, VEGF, EGF, MMP-7/9, uPA, TNF-α and TGF-β, are related to tumors to some extent (Fig. 3, Table 3) [75–77]. Here, we try to identify the influence of TAMs toward the tumor progression through the following aspects (Table 3).

**Table 3:** Roles of TAM-derived cytokines in gynecological and breast cancers

Cytokines	Tumor type	Interacting molecules	Function	References
IL-6	Cervical cancer	IL-6/JAK/STAT3	HPV E6 oncoprotein participates in the phosphorylation activation of the STAT pathway by IL-6 and promotes cancer cell proliferation	[84]
	ER-positive breast cancer		The IL6/STAT3 pathway independent of ER pathway promotes the proliferation of cancer cells	[86]
IL-1	Breast cancer	sCD44	A novel positive feedback loop between IL1β and CD44 promotes TNBC malignant progression	[75]
IGF-1	Breast cancer	Akt	Serum IGF-1 concentrations and IGF-I/IGFBP3 ratio correlated with Akt phosphorylation which promote breast cancer progression and poor disease outcome	[77]
CXCL8/IL-8	Endometrial carcinoma	HOXB13	Down-regulation of ERα expression in endometrial carcinoma, leading to tumor invasion and poor prognosis	[88]
	ER-positive breast cancer	HOXB13, mTOR	Up-regulation of HOXB13 can promote cell proliferation and metastasis in vitro and in vivo by directly inhibiting ERα, while ERα can be inhibited by decreasing the expression of mTOR	[89]
	Epithelial ovarian cancer	Estrogen, IL-6	Estrogen, IL-6 and CXCL8 interact to regulate epithelial ovarian cancer growth through a cascade of amplification effects of ERα pathway	[91, 92]
VEGF-C/D	Endometrial cancer and cervical cancer	VEGFR-3	VEGF-C, VEGF-D and receptor VEGFR-3 are correlated with different stages of tumor progression	[95, 96]
EGF	Ovarian cancer	VEGF	EGFR activation and up-regulation of VEGF/VEGFR signaling pathway in surrounding tumor cells supported tumor cell proliferation and migration. EGF up-regulated αMβ2 integrin and ICAM-1 and promoted the adhesion of tumor cells to TAMs	[99]
MMP-9	ER-positive breast cancer	ER	TAMs secretion of MMP-9 is associated with reduced overall survival via ER	[104]
MMP-7	Ovarian cancer	p38	MMP-7 promoted macrophage infiltration to further form tumor microenvironment	[105]
uPA	Breast ductal carcinoma in situ	uPAR	uPA reshaped the extracellular matrix by degrading the components involved, which in turn led to the removal of tumor cells from the primary site	[110]
TNF-α	Breast cancer	Estrogen	TNF-α involved in EMT and metastasis of breast cancer cells	[76]
IL-10	Cervical cancer	HPV E2 T cell	HPV E2 protein could bind to IL-10 gene promoter, triggering the increase in IL-10. Increased IL-10 inhibited T cell immunity	[118, 119]
	Breast cancer	CD8^+^ T cell	Macrophage-derived IL-10 overexpression leads to tumor development in response to CD8^+^ T cell-dependent chemotherapy	[121, 122]
TGF-β1	Cervical cancer	HPV E6/E7	As tumors develop, TGF-β induces EMT	[118]
	High-grade serous ovarian cancer	Unknown	High levels of TGF-β1 secretion promote peritoneal metastasis of cancer cells	[112]
GDF15	Cervical cancer	PI3K/AKT and MAPK/ERK	The PI3K/AKT and MAPK/ERK signaling pathways are phosphorylated by ErbB2 to promote tumor progression	[113]

#### Promoting tumor cell proliferation

Cancer cells are thought to divide out of control and proliferate indefinitely, making them difficult to be removed and destroying the surrounding normal tissues. Different studies have shown that TAMs secrete IL-6 [78, 79], which is a cytokine belonging to the interleukin family associated with tumorigenesis. It has been proved that IL-6 played a vital role through IL-6-JAK-STAT signal transduction [80–82], and activation of STAT3 was an important factor affecting cell proliferation, differentiation and survival [83]. STAT3 protein level and phosphorylation state were significantly induced in HPV-positive cervical cancer cells compared with HPV-negative ones, which has been demonstrated to be mediated by HPV E6 oncoprotein [84]. Meanwhile, Luan et al. observed a synchronous increase in IL-6R expression of cervical cancer cells, which appears to be detrimental to patients’ prognosis [85]. This suggests that IL-6 secreted by TAMs may affect tumor proliferation, migration and adhesion through up-regulated IL-6R. However, the activation of IL6/STAT3 pathway which was found in a brand-new study promoted the metastasis of ER^+^ intraductal breast cancer in vivo, and it strikingly constituted another oncogenic pathway which was relatively independent of ER pathway [86].

Another member of interleukin family, CXCL8 (also known as IL-8), has been widely concerned in tumorigenesis due to its pluripotency including angiogenesis and mitogenic effects [87]. CXCL8 is mainly secreted by mononuclear macrophages. It has been reported that TAMs down-regulated the expression of ERα in endometrial carcinoma tissues by over-secretion of CXCL8 via homeodomain transcription factor HOXB13, which is associated with tumor invasion and poor prognosis [88]. The parallel results were found in ERα^+^ breast cancer. Up-regulated HOXB13 accelerated cell proliferation and metastasis both in vitro and in vivo through direct suppression of ERα, which could be inhibited by reducing the expression of mTOR [89]. In the meantime, one research found that the expression of ERα in cervical cancer decreased by more than 15 times as cervical cancer progressed from normal to cancer [90]. However, the relationship between ERα reduction and TAMs in cervical cancer cells has not been reported.

Moreover, it has been demonstrated that estrogen, IL-6 and CXCL8 are capable of interacting with each other to regulate the growth of epithelial ovarian cancer through cascade amplification effect via an ERα pathway [91, 92].

#### Dysregulating angiogenesis and lymphangiogenesis

The increasing division and growth of cancer cells indicate that they need more nutrients from the blood. Therefore, tumor angiogenesis plays a pivotal role in solid tumor growth, invasion and metastasis. Recently, TAMs have been shown to tight tie with angiogenesis and lymphangiogenesis through the secretion of VEGF-A, VEGF -C, VEGF-D, EGF, placental growth factor (PlGF), TGF-β, TNF-α, IL-1β, IL-8, CCL2, CXCL8 and CXCL12 [93]. In breast cancer, TAM infiltration is closely associated with VEGF expression and microvascular density (MVD), which jointly affects the prognosis of patients [94]. In addition, previous studies have confirmed that VEGF-C, VEGF-D and their receptor VEGFR-3 were positively correlated with different stages of cervical lesions, allowing the development of lymphangion [95, 96]. Interestingly, the expression of VEGF-C and VEGF-D increased significantly after the disease progressed to CIN3 stage [96]. However, the opposite conclusion has been drawn in endometrial cancer [95]. One study reported that lower level of VEGF-C was detected in endometrial cancer samples [95]. We suspect tha t the char acteristics of different tumors and the limited sample size may account for the controversy.

On the other hand, TAMs expressed HIF-1α to active angiogenesis [97]. In breast cancer, HIF-1α-stabilizing long noncoding RNA (HISLA) was proved to prevent the inactivation of HIF-1α from hydroxylation by prolyl hydroxylase domain 2 (PHD2) [98]. Moreover, HISLA produced by extracellular vesicles released by TAMs can inhibit the interaction between HIF-1 α and PHD2 and promote the stability of HIF-1α in TME [97].

In the study of transcoelomic metastasis of ovarian cancer, TAMs in tumor clusters (spheroids) motivated EGF expression at early stage of metastasis which was integrated with activated tumor EGFR, further supporting tumor cell proliferation and migration by up-regulating VEGF/VEGFR signaling in surrounding tumor cells [99]. EGF was also observed to increase αMβ2 integrin on TAMs and ICAM-1 on tumor cells, promoting the adhesion between TAMs and tumor cells [99]. Ishihara et al. discovered that Wiskott–Aldrich syndrome protein (WASp) allowed TAMs to secrete EGF and approach CSF-1-expressing breast cancer cells chemotactically to regulate tumor metastasis [100].

#### Facilitating EMT and tumor metastasis

EMT and extracellular matrix remodeling constitute remarkable links in promoting tumor metastasis. Jing et al. revealed that under the action of ERα agonists, ERα^+^ M2 macrophages significantly up-regulated CCL18 and activated the mTOR/KIF5B signaling pathway in endometrial cancer cells to promote EMT [101].

Matrix metalloproteinases (MMPs), which are widely presented in TME, can not only facilitate EMT, but also promote lymphangiogenesis in conjunction with the VEGF [93]. Recently, one study demonstrated that M2-like macrophages have been found to secrete large amounts of MMP-9 that promoted tumor progression both in breast cancer and in colon cancer, which was considered as prognostic markers and therapeutic targets [102, 103]. In ER^+^ breast cancer, MMP-9 secreted by TAMs was associated with worse overall survival, but not in ER-negative breast cancer or triple-negative breast cancer (TNBC) [104]. Wen et al. reported that MMP-7 elevation in macrophages which was co-cultured with ovarian cancer cells occurred through the p38 pathway and promoted the infiltration of macrophages to form TME [105]. At the same time, the majority of the studies supported the view that increased MMP-9 can also promote the development of cervical cancer [106, 107], although a few articles have suggested that MMP-9 was helpful for cervical cancer prognosis [103]. Therefore, taking into account the differences in researches, the effect of MMP on cervical cancer needs to be further optimized.

Moreover, TAMs also exert an enormous function in promoting tumor cell metastasis by releasing urokinase-type plasminogen activator (uPA) and up-regulating the expression of uPA receptor (uPAR), which belongs to the fibrinolysis system [108]. The combination of uPA and uPAR initiates the reactions to reshape the extracellular matrix by degrading the relative components and thereafter leads to the distant migration of tumor cells from the primary site [109]. Additionally, increased uPAR can also induce macrophage aggregation to tumor tissue, forming a positive feedback [110]. In breast ductal carcinoma in situ and invasive breast carcinomas, blood monocytes with higher uPAR levels might be selectively recruited into cancer tissues, which induced elevated uPAR levels in TAMs through paracrine action as well [110].

As a member of transforming growth factors, TGF-β is a multifunctional cytokine. It acts as a potent tumor suppressor in the early stage of cancers, while later these cytostatic effects become resistant and TGF-β is involved in inducing EMT [111]. With regard to high-grade serous ovarian cancer, ascites with high levels of TAMs and TGF-β1 facilitated cancer cell migration [112]. In cervical cancer, GDF15 which belongs to TGF-β superfamily phosphorylated PI3K/AKT and MAPK/ERK signaling pathways via ErbB2, a member of EGFR family, to achieve the purpose of promoting tumor progression [113].

#### Forming immunosuppression

Tumor cells escape from recognition and attack by the body's immune system through multitudinous mechanisms. IL-10 is a multicellular cytokine with immunosuppressive effects and can inhibit antigen delivery by the mononuclear phagocyte system [114, 115]. One previous study found that IL-10 promoted the transformation of macrophages into M2-like macrophages in TME, and polarized M2-like macrophages in turn highly secrete IL-10 [116]. In cervical lesions, it has been reported that the amount of IL-10 in TME was associated with the type of HPV infection and the degree of progression from lesions to cervical cancer. Moreover, with the significantly increase in IL-10, the expression of HPV E6 and E7 was detected to augment [117]. Besides, IL-10 has been proven to play a role in anti-tumor T cells [119, 120]. In breast cancer, macrophage-derived IL-10 overexpression resulted in tumor development via inefficiency of CD8^+^ T cell-dependent responses to chemotherapy [121]. As Chu et al. reported, IL-10 was also an independent factor for adverse prognosis in patients with ER-negative breast cancer [122].

In ovarian cancer tissues, Qu et al. discovered the increased expression of PD-L1 on the surface of TAMs, which stimulated apoptosis of T cells to suppress immunity [123]. Up-to-date study has found that overexpression of human epididymis protein 4 (HE4) in ovarian cancer cells may be one of the reasons for the elevated PD-L1 by macrophages [124]. In the meantime, TAMs as well as IFN-γ, TNF-α, IL-10 and especially IL-6 secreted by TAMs induced the expression of PD-L1 both in the cell membrane and in the cytoplasm of ovarian cancer cells, which inhibited the function of CD8^+^ T cells and promoted tumor growth [125]. The same inhibitory effect on T cells was also detected in HPV16-associated cervical cancer through M2 macrophages [119]. Recently, in studies of progression of stromal tumors, the inhibition of CD8^+^ T cells by PD-1/PD-L1 may be realized through the PI3K/Akt/mTOR signaling pathway [126]. Additionally, hypoxic macrophages promote the release of exosomes containing miR-223. It can be transferred to co-cultured epithelial ovarian cancer cells to enhance drug resistance through the PTENPI3K/AKT pathway both in vivo and in vitro [127]. As mentioned above, accompanied by TME hypoxia, VEGF is overexpressed in macrophages. It not only drives angiogenesis, but also suppresses immune cells from playing their normal role [128]. For T cells, VEGF blocks the differentiation of T progenitor cells into CD4^+^ and CD8^+^ lymphocytes, weakens the immune function of T cells. On the other hand, VEGF increases the infiltration of TAMs into tumor sites which forms a positive feedback from VEGF to TAMs [129].

**Fig. 4:**
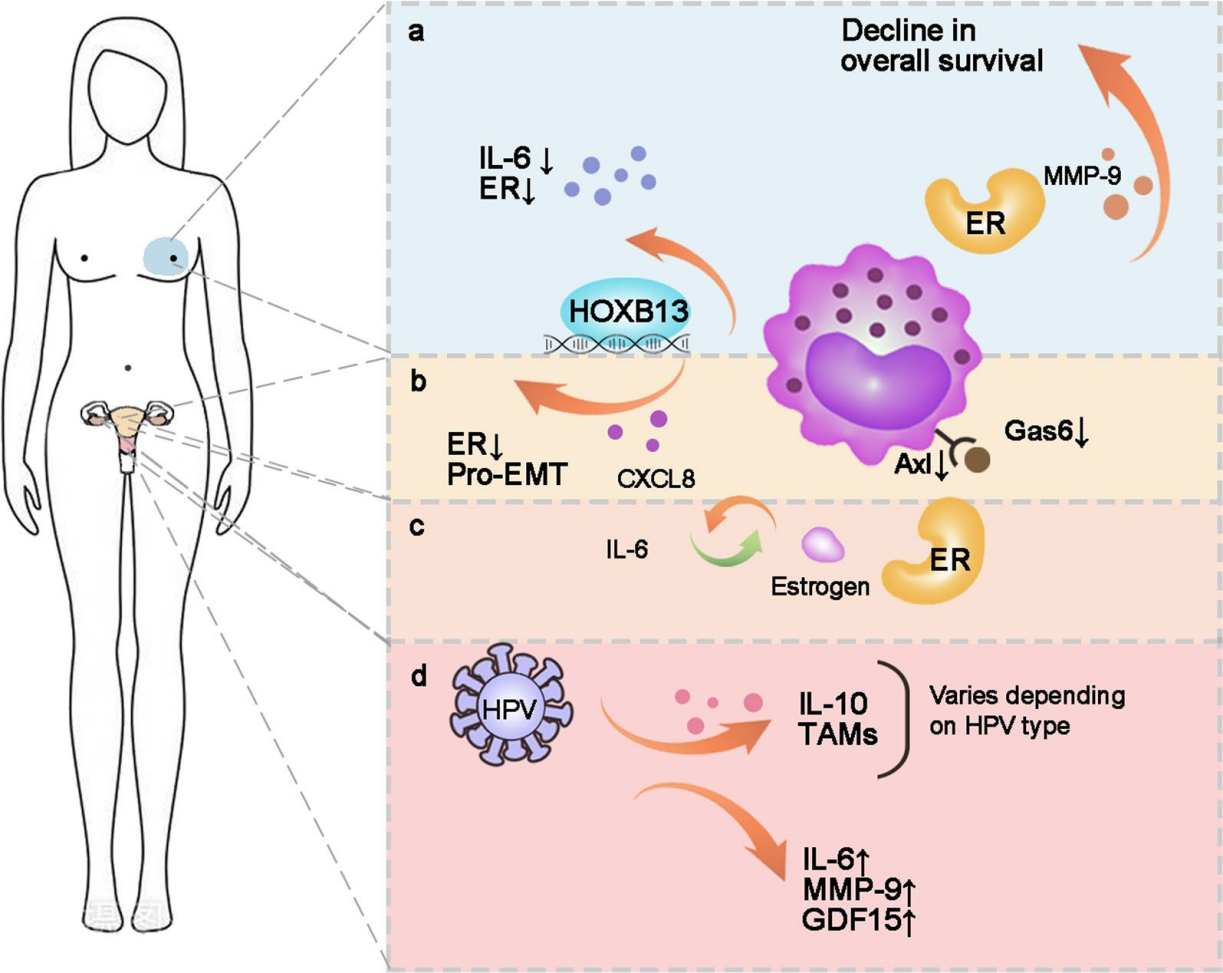
Characteristics of TAMs among gynecological and breast cancers. **a** ER^+^ breast cancer patients had lower survival rates. TAMs up-regulated HOXB13 and down-regulated IL-6 and ER expression. **b** The cascade of estrogen, CXCL8 and IL-6 regulated ovarian cancer cell growth. **c** TAMs promoted EMT in endometrial carcinoma through HOXB13. **d** The type of HPV infection was associated with the number of TAMs and IL-10 expression. TAMs up-regulated the expression of IL-6, MMP-9 and GDF15 in cervical cancer

Taken together, TAMs are involved in the secretion of cytokines and growth factors to influence tumor growth, metastasis and progression. Different types of gynecological and breast cancers have similar or specific characteristics of TAMs (Fig. 4). Comprehensive studies need to be carried out to find more factors secreted by TAMs which take part in tumor regulation, providing more insight into targeted treatments toward TAMs.

### TAM-targeted therapy in gynecological and breast cancers

**Fig. 5:**
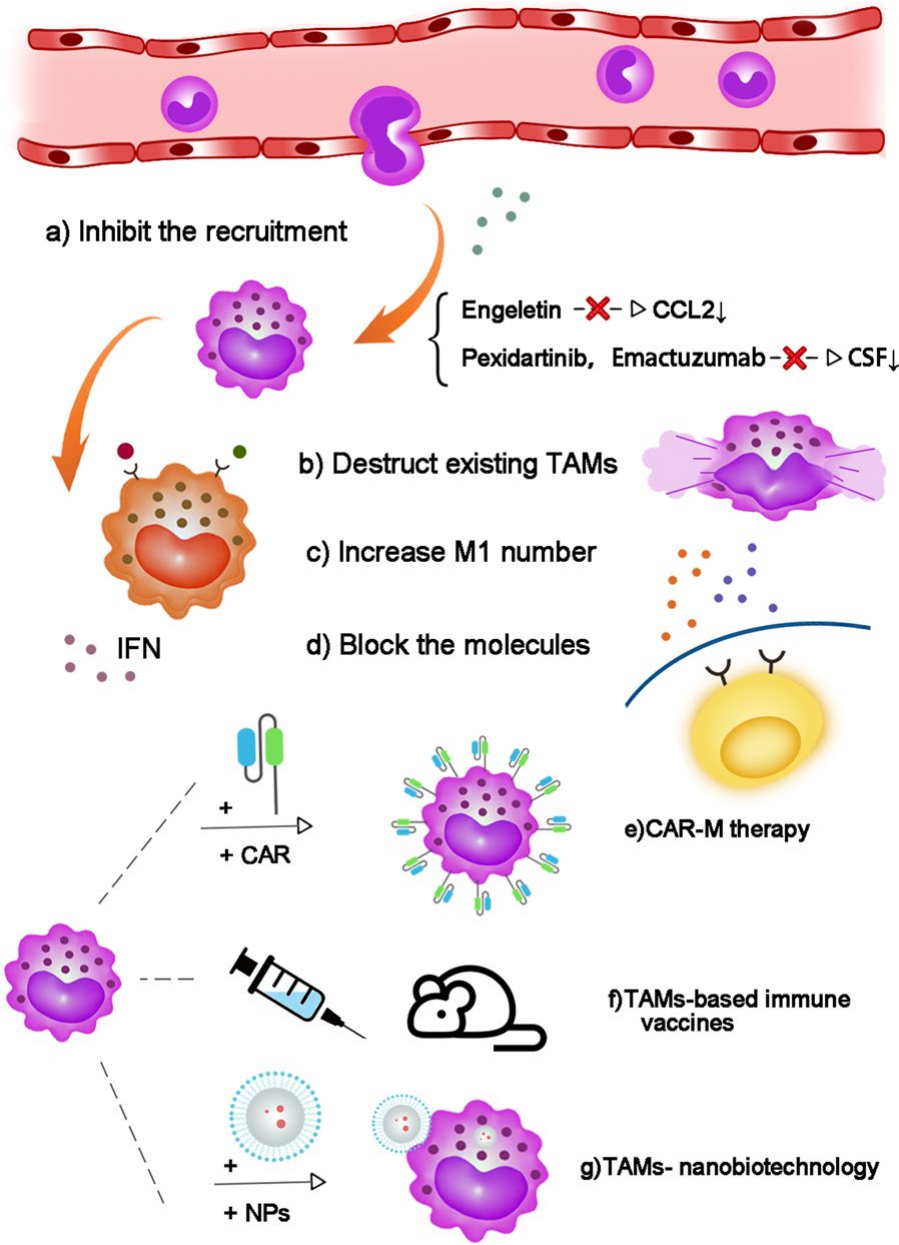
Potential targeted therapeutic strategies fo r TAMs. **a** Directly blocking macrophage chemokines or inhibiting their receptors. **b** Using clodronate to deplete macrophages. **c** Increasing the quantity of M1 or promoting the M2-to-M1 conversion to lift the ratio of M1/M2. **d** Blocking TAMs secretions acting on cancer cells. **e** CAR macrophage therapy. **f** TAMs-based immune vaccine s. **g** TAMs nanobiotechnology

Macrophages are a kind of immune cells that can be stimulated by TME to display different characteristics and have plentiful functions in cancer proliferation, metastasis and immune resistance. Nowadays, various clinical trials on TAM-targeted therapy have been carried out in gynecological and breast cancers (https://clinicaltrials.gov/, Table 4). After the above discussion on the roles of TAMs, we are deeply aware that the intervention of TAMs including depleting macrophages, inhibiting pro-TAMs or activating anti-TAMs, might be a promising therapeutic approach (Fig. 5, Table 5).

**Table 4:** Registered clinical trials involving targeting TAMs in gynecological and breast cancers

TAM target	NCT Number	Study results	Conditions	Interventions	Phases	Year of registration
CAR-M	NCT04660929	NA	Breast cancer Ovarian epithelial cancer	Biological: CT-0508	Phase 1	2020
CD47	NCT04881045	NA	Ovarian cancer	Biological: PF-07257876	Phase 1	2021
	NCT03957096	NA	Breast cancer Ovarian cancer	Drug: SGN-CD47M	Phase 1	2019
	NCT02890368	NA	Breast cancer HPV-related malignant neoplasm	Drug: TTI-621 monotherapy Drug: TTI-621 + PD-1/PD-L1 Inhibitor Drug: TTI-621 + pegylated interferon-α2a Other: TTI-621 + T-Vec Other: TTI-621 + radiation	Phase 1	2016
	NCT02125344	NA	Breast cancer	Drug: non-pegylated liposomal doxorubicin Drug: Carboplatin Drug: Paclitaxel Drug: Epirubicin Drug: Cyclophosphamide Drug: Pertuzumab Drug: Trastuzumab Drug: Ferric carboxymaltose	Phase 3	2014
TLR	NCT04278144	NA	HER2-positive solid tumors	Drug: BDC-1001 Drug: Pembrolizumab	Phase 1/2	2020
	NCT04116320	NA	Breast cancer Cervical cancer Ovarian cancer	Device: Echopulse Drug: Imiquimod Drug: Standard of care PD-1 therapy	Phase 1	2019
	NCT01421017	NA	Breast cancer Metastatic breast cancer Recurrent breast cancer	Radiation: Radiation Drug: Imiquimod Drug: Cyclophosphamide	Phase 1/2	2011
	NCT00899574	2 patients achieved a partial response (20%; 95% CI, 3%-56%)	Breast cancer Breast neoplasms	Drug: Imiquimod	Phase 2	2009
	NCT00319748	0 complete response, 0 partial response, 1 stable disease, 2 progressive disease	Breast cancer Ovarian cancer Endometrial cancer Cervical cancer	Drug: 852A	Phase 2	2006
CSF	NCT04542356	NA	Cervical cancer	Drug: PEG-rhG-CSF Drug: rhG-CSF	Phase 2	2020
	NCT04514692	NA	Cervix cancer Endometrial cancer	Diagnostic Test: FDG PET/CT Drug: GCSF	Phase 1/2	2020
	NCT04418219	NA	Breast cancer	Drug: Cyclophosphamide Biological: Allogeneic GM-CSF-secreting Breast Cancer Vaccine SV-BR-1-GM Biological: Pembrolizumab Biological: Recombinant Interferon Alpha 2b-like Protein Other: Questionnaire Administration Other: Quality of Life Assessment	Phase 1/2	2020
	NCT04144023	NA	Breast ductal carcinoma in situ	Biological: Granulocyte-macrophage colony-stimulating factor Biological: Multi-epitope HER2 peptide vaccine H2NVAC Procedure: Therapeutic conventional surgery	Phase 1	2019
	NCT03858166	NA	Ovarian neoplasms Ovarian cancer	Drug: PEG-rhG-CSF	Phase 4	2019
	NCT03206684	NA	Cervical cancer	Drug: PEG-rhG-CSF Drug: rhG-CSF	Phase 4	2017
	NCT02978222	NA	Platinum-sensitive ovarian cancer Ovarian cancer	Biological: FRα peptide plus Adjuvant (GM-CSF) Drug: Adjuvant (GM-CSF) Alone	Phase 2	2016
	NCT02469116	16 complete response, 1 partial response, 0 stable disease, 1 progressive disease	Ovarian cancer	Drug: Docetaxel Drug: Carboplatin Drug: Pegylated G-CSF	Phase 2	2016
	NCT02017678	NA	Ovarian cancer	Biological: JX-594	Phase 2	2013
	NCT01570036	DFS was improved for NeuVax versus control (HR, 0.26; 95% CI, 0.08–0.81, *P* = 0.01)	Breast cancer	Drug: Herceptin Drug: NeuVax vaccine Drug: GM-CSF (control)	Phase 2	2012

**Table 5:** Strategies of targeted therapy for TAMs

Strategy	Method	Function	References
To reduce TAMs generation	Clodronate-containing liposomes (CL) Engeletin, CSF1/CSF1R inhibitors (MCS110, Pexidartinib and Emactuzumab), cox-oxidase inhibitors	CL can deplete macrophages in vivo and inhibitors of chemokines can reduce TAMs recruitment	[39, 43, 119, 131, 132]
To reactivate M1 TAMs phagocytosis	IFN-γ/IDO1 Anti-CD47 antibodies (Hu5F9-G4, HX009, AK117) HMGB1 inhibitors (A Box, RAP and EP), TGF-β receptor I inhibitors (SB4x), DNMTi 5-Azacytidine (5AZA-C) and ornithine decarboxylase inhibitor α-difluoromethylchlorine (DFMO) TAM receptors inhibitors (BMS-777607, BGB324 and A VB-S6-500)	Targeting IFN-γ/IDO1 pathway, blocking CD47-SIRPα axis and using several valid drugs can reactivate macrophage phagocytosis and convert M2 into M1 macrophages Blocking TAM receptors can inhibit TAM activation and polarization	[142, 143–145, 160, 170–172]
To inhibit macrophage-derived molecules	Broad-spectrum MMP inhibitor (CP-471,474) Selective MMP-12 inhibitors (CGA, CGA-1 and AGA) uPA inhibitor B-428 IL-6 inhibitor Siltuximab, Tocilizumab and Cetuximab TGF-β inhibitor Fresolimumab (GC1008), LY3022859, LY2109761 and 1D11 IL-10 receptor-blocking mAb (αIL-10R; clone 1B1.3A)	Inhibitors of TAMs-derived molecules (MMPs, uPA, IL-6, TGF-β, IL-10) were used to block the occurrence and development of tumor cells	[121, 177–184, 186, 189, 190]
CAR macrophage therapy	Edited specific chimeric antigen receptor on macrophages to target antigen (such as CD19, HER2)	CAR-M therapy can reverse M2 into M1 macrophages and activate phagocytosis	[169, 192, 193]
TAMs-based immune vaccines	Fused macrophage–tumor cytomembrane with vaccine adjuvant (CpG ODNs) Therapeutic vaccine with TAMs receptor inhibitor	TAMs-based immune vaccines can increase M1 macrophages and enhance immune response of immune cells. Combining therapeutic vaccine with TAMs receptor inhibitor obtains synergistic therapeutic effects	[191, 194]
TAMs nanobiotechnology	Macrolide-Au nanorod aCD47@CaCO3 NPs R848-loaded NPs Supramolecular NPs	Nanobiotechnology can target TAMs to boost anti-tumor effect, release CD47, or active TLR-7/TLR-8 to amplify M1 repolarization and phagocytosis	[196, 200]

#### Reducing TAMs generation

The polarization of TAMs is regulated by a variety of microenvironmental cytokines, growth factors and signals from both tumor and stromal cells, which is a process of mutual conversion between M1 and M2 [14]. Considering the extensive role of TAMs in TME, controlling the number of TAMs, in other words, suppressing the polarization of M2 macrophages or guiding macrophages to differentiate into M1-like have wide interest as a new treatment strategy.

In terms of reducing the number of TAMs, clodronate has emerged as the primary choice for drugs to deplete macrophages [130]. In cervical cancer, Lepique et al. injected clodronate-containing liposomes (CL) in a mouse model to consume macrophages by accumulating methylene-containing ATP analogues in the cytoplasm. Although CL did not directly regulate the cytoactive of cancer cells, the reduced number of macrophages inhibited tumor growth in vivo and contribute to the infiltration of tumors by HPV16 E7(49–57)-specific CD8 lymphocytes [119]. However, the therapy still needs further optimization to meet the clinical needs, for example, how to focus on TAMs without interfering with other macrophages in normal body tissues.

In another way, impeding the recruitment of TAMs into tumor tissue has broad application prospects in the future by preventing TAMs recruitment through cytokines. Engeletin (ENG, dihydrokaempferol 3-rhamnoside) is a substance which has anti-inflammatory properties [131, 132]. In cervical cancer, one research found that ENG significantly reduced the secretion of CCL2 via blocking NF-κB signaling pathway, which could be considered as an inhibitor of NF-κB [133]. Due to the help of ENG, monocytes in peripheral blood were less recruited to TME. CSF1/CSF1R inhibitors such as MCS110, Pexidartinib (PLX3397, PLX108-01) and Emactuzumab (RG7155) are being studied in clinical trials in solid tumors including ovarian and endometrial cancers [43, 134]. Heusinkveld et al. reported that a certain concentration of PGE2 and IL-6 could induce CD14^+^ macrophages to differentiate into CD163^+^ M2 macrophages [34]. Fortunately, the effect of PGE and IL-6 was completely blocked by the cox-oxidase inhibitor and IL-6 antibodies [34]. Similarly, the use of inhibitors of various pro-factors such as IL-4, IL-10, TGF-β and IL-13 may reduce the migration of TAMs toward the tumor microenvironment to a certain extent [135–137].

In cervical cancer, compared with HPV preventive vaccine which has been already universally in use as a countermeasure of HPV-associated cervical cancer [138], the therapeutic HPV vaccine is still in development. In a vaccine trial involving the HPV16 E7 43–77 peptide, a decrease was observed in the number of M2 macrophages and the expression of M2 macrophage-related cytokines, such as IL10, TGF-β, MMP-2, MMP-9 and VEGF in the vaccine group [139], while M1 macrophage-related chemokines (such as CXCL-9 and CXCL-10) were up-regulated [139].

#### Reactivating M1 TAMs phagocytosis

As mentioned above, low M1/M2 rate often predicts poor patients’ prognosis [140]. Therefore, trying to increase the number of M1 or improve the ratio of M1/M2 is a feasible way for possible treatment [141]. Indoleamine-2,3-dioxygenase (IDO1) is a key enzyme to degrade amino acid tryptophan through the kynurenine pathway [142], which can regulate macrophages and inhibit the immune function of T cells in a variety of diseases and cancers [142–145]. In cervical cancer, existing studies have shown that IFN-γ activated autophagy of cervical cancer cells and macrophage phagocytosis reactivation through IDO1 overexpression and kynurenine metabolism [146, 147]. In addition to cervical cancer, Feng et al. confirmed that high expression of IDO1 showed better survival prognosis in breast and ovarian cancer [148]. Although these studies revealed that up-regulation of IDO1 was beneficial to the suppression of cancer to some degree, more studies indicated that overexpressed IDO1 was related to immunosuppression and played an adverse role in the process of tumor development [149, 150].

Cancer cells express CD47 to protect cells from phagocytosis by binding and activating the receptor SIRPα on macrophages to inhibit macrophage phagocytosis. Targeting CD47-SIRPα axis has become a potential therapeutic mechanism in various preclinical models by stimulating the phagocytosis of cancer cells in vitro and in vivo [151, 152]. Up-regulated expression of CD47 was found in almost all types of tumor cells, which was associated with poor survival and prognosis [153–155]. Huang et al. constructed a novel oncolytic adenovirus carrying a signal regulatory protein-α (SIRPα)-IgG1 Fc fusion gene (termed SG635-SF), which significantly inhibited the growth of ovarian cancer cells in vivo [156]. Samanta et al. reported that treatment of TNBC cells with carboplatin, doxorubicin, gemcitabine or paclitaxel promoted the enrichment of CD47^+^CD73^+^PDL1^+^ TNBC cells and promoted tumor immune evasion, which could be eliminated by inhibiting HIF [157]. Based on the crucial role of CD47-SIRPα axis in tumor immune escape, clinical trials of anti-CD47 antibodies are under way, involving drugs such as Hu5F9-G4, HX009 and AK117. A breast cancer study found that CD47 and HER2 activated each other at the transcriptional level [158]. Double antibody blocking CD47 and HER2 not only inhibited tumor clone formation, but also enhanced macrophage-mediated attack [158]. In TNBC, anti-CD47 antibody inhibited the differentiation of cancer stem cells (CSC) and down-regulated the expression of EGFR [159]. In addition, immunoliposome (ILips) combined with anti-CD47 and paclitaxel also produced a synergistic anti-tumor effect on converting M2 to M1 and enhancing systemic T cell immune response [160].

High-mobility group box 1 (HMGB1) is a non-histone chromatin-binding protein that exists widely in the nucleus and cytoplasm, which has been proven to up-regulate Treg cells and M2 macrophages in basal-like breast cancer cells [161]. Moreover, this effect could be blocked by HMGB1 inhibitors A Box, RAP, or EP, resulting in a reduced proportion of Treg cells and an up-regulated M1/M2 ratio [161]. Similarly, in the study of traditional Chinese medicine (TCM) treatment for ovarian cancer, astragaloside IV was found to weaken the polarization of M2 macrophages by inhibiting HMGB1-TLR4 signal transduction [162].

Toll-like receptors (TLRs) refer to conserved membrane proteins that recognize pathogens [163]. A study verified that TLR7 bound with TGF-β receptor I inhibitors SB431542 (SB4x) jointly provoked the polarization of M1 and CD4^+^, CD8^+^ and CD19^+^ cells [164]. Travers et al. found that DNMTi 5-azacytidine (5AZA-C) activated type I interferon signaling in ovarian cancer, catalyzed the number of IFN-γ^+^ immune cells and lowered the percentage of macrophages in TME [165]. Furthermore, 5AZA-C combined with the ornithine decarboxylase inhibitor α-difluoromethylchlorine (DFMO) significantly reduced the M2 macrophages and increased the M1 macrophages [165]. Zhao et al. discovered that colorectal cancer treated with Cetuximab repolarized TAMs from M2 to M1 macrophages in an IL6- dependent manner [166].

In the above description of biomarkers for TAMs, we mentioned that stimulating macropha ges with different factors can affect the expression of receptors (Tyro3, Axl and MerTK receptor) on the surface of macrophages. Sun et al. proved that the expressions of Axl and its ligands Gas6 were abnormal in highly differentiated endometrial cancer, which may be related to tumor progression and inhibition of apoptosis [168]. However, Goyette et al. found that high AxL protein levels were associated with tumor metastases in HER2^+^ breast cancer, which was independent of Gas6 [169]. Taking into account that TAMs receptors mediate tumor growth, invasion and metastasis, the researches on receptor inhibitors are booming [167]. Several studies found that pan TAMs kinase inhibitor BMS-777607 could inhibit the activation of TAMs receptor, further block the PD-L1 signaling pathway and suppress tumor progression [170], while selective Axl inhibitor BGB324 appears to induce apoptosis by inhibiting the PI3K pathway [171]. In addition, the function of AVB-S6-500 has been confirmed in clinical trials as an anti-Axl Fc fusion protein in epithelial ovarian cancer, primary peritoneal cancer and fallopian tube cancer [172].

Additionally, Zhao et al. demonstrated that promoting the expression of long noncoding RNA (lncRNA)-Xist in M1 macrophages and down-regulating microRNA (miR)-101 expression in M2 macrophages could facilitate M2-to-M1 macrophage-type conversion, which might play a beneficial role in inhibiting the proliferation and migration of breast and ovarian cancers [173].

#### Inhibiting macrophage-derived cytokines

As previously stated, macrophages promote the growth and metastasis of tumor cells by secreting cytokines and growth factors. Therefore, blocking the corresponding signal transduction to interfere with cytokines is another method to restrict the role of TAMs.

Recent studies have shown that in cervical cancer, Poly(I:C), an adjuvant for cancer vaccine, may act through the NF-κB signaling pathway to promote the expression of M1-type cytokines (such as IL-1β and IL-6) and inhibit the expression of M2-type cytokines (such as IL-10 and CCL22) [174]. Another study showed that TAMs autocrine VEGF also acted through NF-κB pathway [175]. Blocking the transmission of TAMs internal NF-κB pathway can weaken the pro-angiogenesis effect of VEGF as well. Besides, in MMP-12 knockout macrophages, the expression and phosphorylation of p38 and ERK1/2 were reduced, and the secretions of IL-1β, IL-6, TNF-α, CXCL1 and CXCL3 were down-regulated [176]. Three novel selective inhibitors of hydroxy ester MMP-12 (CGA, CGA-1 and AGA) and two 99mTc radioactive tracers derived from AGA [99mTc]-AGA-1 and [99mTc]-AGA-2 have been reported in recent studies [177]. In addition, a broad-spectrum MMP inhibitor CP-471/474 and a dual MMP-9/MMP-12 inhibitor (AZ11557272) showed well inhibition of MMP [178, 179]. However, further clinical trials are needed for selective MMP inhibitors to reduce drug toxicity. As for uPA, its inhibitor, 4-iodo benzo [b]thiophene-2-carboxamidine (B-428), has been shown to hinder the growth and metastasis of breast cancer in combination with tamoxifen back since the 1990s, which was superior to their separative effects [180].

Based on the important role of TAMs-derived IL-6 in cancer, therapeutic strategies targeting the IL-6 pathway are in active development. Siltuximab pertains to monoclonal antibodies (Mabs) that block IL-6 receptor [181]. In Siltuximab-treated ovarian cancer and ERα-positive breast cancer, cancer cells were found to reduce IL-6 production, which inhibited tumor growth, TAMs invasion and angiogenesis [182–184]. Analogously, Tocilizumab was effective in ovarian cancer, cervical cancer, endometrial cancer and breast cancer [185–188]. Besides, one study discovered that Cetuximab could also enhance the anti-tumor function of macrophages through IL-6 pathway [166].

To deal with up-regulated TGF-β, the researchers used antibodies to block both the receptor and the ligand, such as Fresolimumab (GC1008) and LY3022859 [189]. Altering the structure or composition of the ligand or the receptor to affect their binding is also helpful [189]. TGF-β targeting agents, such as LY2109761 and 1D11, have been proven effective in inhibiting metastasis in metastatic breast cancer [190].

IL-10 is an attractive therapeutic target due to its immunosuppression function and participation in tumorigenesis. One previous experiment showed that IL-10 receptor-blocking mAb (αIL-10R; clone 1B1.3A) increased the therapeutic effect of chemotherapy in breast cancer model in a CD8^+^ T cell-dependent manner [121]. Additionally, Alotaibi et al. used RNAi to reduce secretion of IL-10, resulting in accelerating the apoptosis of breast cancer cells with a suppression in PI3K/Akt pathway [136].

To sum up, therapy targeting cytokines and growth factors can be performed in the following ways. Firstly, at the gene level, RNA interference technology can influence molecule expression. Besides, the expression of genes can be blocked by interfering the normal signal transducing pathway. In addition, we can also use a variety of methods to block the binding of receptors and ligands and further block the role of ligands.

#### CAR-macrophage (CAR-M) therapy

In recent years, chimeric antigen receptor (CAR) T cell therapy has developed rapidly in the treatment of hematological tumors. At the same time, human macrophages, which are modified with edited specific CAR to target antigen (such as CD19 and HER2) in order to recognize tumor cells and improve the ability of phagocytosis and antigen presentations, are in development as an immunotherapy [191]. The application of CAR-M therapy in solid tumors was first reported by Klichinsky et al. in 2020 [192]. The study has shown that CAR-M targeting HER2 significantly reduced tumor load, inhibited lung metastasis and prolonged the survival time in ovarian cancer mouse model [192]. Moreover, the expression of CAR structure can also promote TAMs polarization and even reverse M2 macrophages to transform into M1 macrophages [192, 193]. In addition, CAR-expressing macrophage cells (CAR-iMac) based on induced pluripotent stem cells (iPSCs) showed the ability to inhibit tumor cell growth both in hematological and in ovarian cancer model with lift expression of M1 pro-inflammatory cytokines [193].

#### TAMs-based immune vaccines

As a novel strategy, tumor immune vaccines based on macrophages can reshape the TME and prevent tumor recurrence after operation. Wang et al. designed a biologically fused cytomembrane (FM) which contained both M1 macrophage and 4T1 breast tumor cell cytomembrane [194]. With the assistance of vaccine adjuvant cytosine-phosphate-guanosine oligodeoxynucleotides (CpG ODNs), the vaccine successfully increased the number of pro-inflammatory M1 macrophages, promoted the maturation of dendritic cells and activated memory T cells [194]. On the other hand, the combined vaccine which consists of therapeutic vaccine and TAMs receptor inhibitor provides a new way to treat cancer by inhibiting the up-regulated TAM receptors after vaccine treatment [191].

#### TAMs-nanobiotechnology

Nanobiotechnology targeting TAMs is another promising immunotherapy. Nanoparticles loaded with TLR agonists, cytokines, monoclonal antibodies and metal oxides have achieved preferable results in cancer therapy [195, 196]. CAR-M combined with nanocarrier has been proved to be effective in solid tumors [197]. Kang et al. treated monocytes or macrophages with genes encoding CAR and IFN-γ through polymer nanocarriers, which successfully led to the phenotypic transition from M2 to M1 and the enhancement of macrophage phagocytosis [197]. In breast cancer model, TAMs were modified by iron-based metal organic framework (Fe-MOF) nanoparticles with ferroptosis-inducing agents, which allowed iron ions released in TAMs and further enhanced ferroptosis-associated stress in macrophages to induce effective M1 phenotypic polarization [195]. TAM-membrane-coated nanoparticles with conjugated photosensitizer (NPR@TAMM) and CSFR not only consumed tumor-derived CSF1, but also induced M2 macrophages transform into M1 via photodynamic immunotherapy [198]. Besides, nanoparticles loaded with TLR-7/TLR-8 agonist resiquimod (R848) and doxorubicin (DOX), which induced immunogenic cell death (ICD) or loaded with curcumin (Cur) and baicalin (Bai), had satisfactory tumor cytotoxicity in TME and significantly improved the efficacy of immunotherapy [199, 200].

## Conclusions

It is clear that TAMs in gynecological and breast cancers affect tumor proliferation, angiogenesis, metastasis and immunosuppression by secreting cytokines and expressing receptors, which is related to the amount and proportion of anti-tumor M1 and pro-tumor M2 in TME. Since the phenotype of TAMs is elusive, scientists have explored the biomarkers of TAMs in attempt to distinguish TAMs and relevant cytokines, hoping to provide new ways to conquer tumors.

However, TAMs-targeted therapy has not yet achieved total success. To realize its full potential, several issues need to be addressed. First, complex mechanisms of TAMs in TME formation have not been fully clarified. Secondly, the accuracy of target tumor-promoting TAMs is the main problem facing various targeted drugs, and drug resistance and side effects should also be taken into consideration. In addition, the specificity of gynecological and breast cancers may contribute to different cancer-promoting patterns of TAMs via different gene expression and mechanistic pathways.

In  conclusion, the relationship between TAMs and gynecological and breast cancers is worth in-depth investigation. Future researches could focus on new tumor-promoting mechanisms and targeted therapies including TAMs-based immunotherapies. There is no doubt that targeting TAMs in combination with existing therapies may improve patient outcomes in the future, which emerges as a novel treatment approach with great potential.

## Data Availability

Not applicable.

## Abbreviations

TME: Tumor microenvironment
TAM: Tumor-associated macrophage
M1: M1-like macrophages
M2: M2-like macrophages
EMT: Epithelial mesenchymal transformation
OSM: Oncostatin M
mTORC2: mTOR signaling complex 2
CD: Cluster of differentiation
CL: Clodronate-containing liposomes
TLRs: Toll-like receptors
Gas6: Growth inhibition specificity 6
HPV: Human papillomavirus
ER: Estrogen receptor
PlGF: Placental growth factor
MVD: Microvascular density
WASp: Wiskott–Aldrich syndrome protein
MMPs: Matrix metalloproteinases
TNBC: Triple-negative breast cancer
uPA: Urokinase-type plasminogen activator
PHD2: Prolyl hydroxylase domain 2
ENG: Engeletin
Treg: Regulatory T
MSC: Mesenchymal stem cells
MDSCs: Myeloid-derived suppressor cells
TRMs: Tissue-resident macrophages
BMDMs: Bone marrow-derived macrophages
CM: Conditioned medium
MVD: Microvascular density
TCM: Traditional Chinese medicine
CAR: Chimeric antigen receptor

## References

[1] Sung H, Ferlay J, Siegel RL (2021). Global cancer statistics 2020: GLOBOCAN estimates of incidence and mortality worldwide for 36 cancers in 185 countries. CA Cancer J Clin.

[2] Liu Y, Li L, Li Y, Zhao X (2020). Research progress on tumor-associated macrophages and inflammation in cervical cancer. Biomed Res Int.

[3] Kim J, Bae JS (2016). Tumor-associated macrophages and neutrophils in tumor microenvironment. Mediators Inflamm.

[4] Petty AJ, Yang Y (2017). Tumor-associated macrophages: implications in cancer immunotherapy. Immunotherapy.

[5] Oyervides-Muñoz MA, Pérez-Maya AA, Rodríguez-Gutiérrez HF (2018). Understanding the HPV integration and its progression to cervical cancer. Infect Genet Evol.

[6] Castellaro AM, Rodriguez-Baili MC, Di Tada CE, Gil GA (2019). Tumor-associated macrophages induce endocrine therapy resistance in ER+ breast cancer cells. Cancers (Basel).

[7] Lindsten T, Hedbrant A, Ramberg A (2017). Effect of macrophages on breast cancer cell proliferation, and on expression of hormone receptors, uPAR and HER-2. Int J Oncol.

[8] Zhang J, Zhang Q, Yang Y, Wang Q (2020). Association between succinate receptor SUCNR1 expression and immune infiltrates in ovarian cancer. Front Mol Biosci.

[9] Zhang Q, Wang Q, Wu S, Zhang J (2020). Clinical implication and immunological characterisation of the ARF-GEF family member CYTH4 in ovarian cancer. Autoimmunity.

[10] Wang Q, Zhang Q, Li Q et al. Clinicopathological and immunological characterization of RNA m(6) A methylation regulators in ovarian cancer. Mol Genet Genom Med. 2021; 9: e1547. 10.1002/mgg3.1547 PMC7963423 33225598

[11] Kim SY, Nair MG (2019). Macrophages in wound healing: activation and plasticity. Immunol Cell Biol.

[12] Condeelis J, Pollard JW (2006). Macrophages: obligate partners for tumor cell migration, invasion, and metastasis. Cell.

[13] Ostuni R, Kratochvill F, Murray PJ, Natoli G (2015). Macrophages and cancer: from mechanisms to therapeutic implications. Trends Immunol.

[14] Lin Y, Xu J, Lan H (2019). Tumor-associated macrophages in tumor metastasis: biological roles and clinical therapeutic applications. J Hematol Oncol.

[15] Güç E, Pollard JW (2021). Redefining macrophage and neutrophil biology in the metastatic cascade. Immunity.

[16] Chen P, Zuo H, Xiong H (2017). Gpr132 sensing of lactate mediates tumor-macrophage interplay to promote breast cancer metastasis. Proc Natl Acad Sci USA.

[17] Mu X, Shi W, Xu Y (2018). Tumor-derived lactate induces M2 macrophage polarization via the activation of the ERK/STAT3 signaling pathway in breast cancer. Cell Cycle.

[18] Jia X, Yu F, Wang J (2014). Emodin suppresses pulmonary metastasis of breast cancer accompanied with decreased macrophage recruitment and M2 polarization in the lungs. Breast Cancer Res Treat.

[19] Weng YS, Tseng HY, Chen YA (2019). MCT-1/miR-34a/IL-6/IL-6R signaling axis promotes EMT progression, cancer stemness and M2 macrophage polarization in triple-negative breast cancer. Mol Cancer.

[20] Meng Z, Zhang R, Wang Y et al. miR-200c/PAI-2 promotes the progression of triple negative breast cancer via M1/M2 polarization induction of macrophage. Int Immunopharmacol 2020; 81:106028 10.1016/j.intimp.2019.106028 31801690

[21] Bai Y, Yin K, Su T (2020). CTHRC1 in ovarian cancer promotes M2-like polarization of tumor-associated macrophages via regulation of the STAT6 signaling pathway. Onco Targets Ther.

[22] Deng X, Zhang P, Liang T (2015). Ovarian cancer stem cells induce the M2 polarization of macrophages through the PPARγ and NF-κB pathways. Int J Mol Med.

[23] Zhou YX, Zhao W, Mao LW (2018). Long non-coding RNA NIFK-AS1 inhibits M2 polarization of macrophages in endometrial cancer through targeting miR-146a. Int J Biochem Cell Biol.

[24] Li LY, Yin KM, Bai YH (2019). CTHRC1 promotes M2-like macrophage recruitment and myometrial invasion in endometrial carcinoma by integrin-Akt signaling pathway. Clin Exp Metastasis.

[25] Li Q, Liu L, Zhang Q (2014). Interleukin-17 indirectly promotes M2 macrophage differentiation through stimulation of COX-2/PGE2 pathway in the cancer cells. Cancer Res Treat.

[26] Wu JY, Huang TW, Hsieh YT (2020). Cancer-derived succinate promotes macrophage polarization and cancer metastasis via succinate receptor. Mol Cell.

[27] Cheng Y, Zhu Y, Xu J (2018). PKN2 in colon cancer cells inhibits M2 phenotype polarization of tumor-associated macrophages via regulating DUSP6-Erk1/2 pathway. Mol Cancer.

[28] Cheng Y, Zhu Y, Xu W (2018). PKCα in colon cancer cells promotes M1 macrophage polarization via MKK3/6-P38 MAPK pathway. Mol Carcinog.

[29] Lian G, Chen S, Ouyang M (2019). Colon cancer cell secretes EGF to promote M2 polarization of TAM through EGFR/PI3K/AKT/mTOR pathway. Technol Cancer Res Treat.

[30] Huang C, Ou R, Chen X (2021). Tumor cell-derived SPON2 promotes M2-polarized tumor-associated macrophage infiltration and cancer progression by activating PYK2 in CRC. J Exp Clin Cancer Res.

[31] Li Q, Wu W, Gong D (2021). Propionibacterium acnes overabundance in gastric cancer promote M2 polarization of macrophages via a TLR4/PI3K/Akt signaling. Gastric Cancer.

[32] Pathria P, Louis TL, Varner JA (2019). Targeting tumor-associated macrophages in cancer. Trends Immunol.

[33] Noy R, Pollard JW (2014). Tumor-associated macrophages: from mechanisms to therapy. Immunity.

[34] Heusinkveld M, de Vos van Steenwijk PJ, Goedemans R et al. M2 macrophages induced by prostaglandin E2 and IL-6 from cervical carcinoma are switched to activated M1 macrophages by CD4+ Th1 cells. J Immunol. 2011;187:1157–65. 10.4049/jimmunol.1100889 21709158

[35] Nakamura K, Smyth MJ (2020). Myeloid immunosuppression and immune checkpoints in the tumor microenvironment. Cell Mol Immunol.

[36] De Nola R, Menga A, Castegna A et al. The crowded crosstalk between cancer cells and stromal microenvironment in gynecological malignancies: biological pathways and therapeutic implication. Int J Mol Sci. 2019; 20. 10.3390/ijms20102401 PMC6567055 31096567

[37] Murray PJ, Allen JE, Biswas SK (2014). Macrophage activation and polarization: nomenclature and experimental guidelines. Immunity.

[38] Chen Y, Song Y, Du W (2019). Tumor-associated macrophages: an accomplice in solid tumor progression. J Biomed Sci.

[39] Chávez-Galán L, Olleros ML, Vesin D, Garcia I (2015). Much more than M1 and M2 macrophages, there are also CD169(+) and TCR(+) macrophages. Front Immunol.

[40] Wang Q, Steger A, Mahner S (2019). The formation and therapeutic update of tumor-associated macrophages in cervical cancer. Int J Mol Sci.

[41] Pedraza-Brindis EJ, Sánchez-Reyes K, Hernández-Flores G (2016). Culture supernatants of cervical cancer cells induce an M2 phenotypic profile in THP-1 macrophages. Cell Immunol.

[42] Kawamura K, Komohara Y, Takaishi K (2009). Detection of M2 macrophages and colony-stimulating factor 1 expression in serous and mucinous ovarian epithelial tumors. Pathol Int.

[43] Hua F, Tian Y, Gao Y (2019). Colony-stimulating factor 1 receptor inhibition blocks macrophage infiltration and endometrial cancer cell proliferation. Mol Med Rep.

[44] Sullivan AR, Pixley FJ (2014). CSF-1R signaling in health and disease: a focus on the mammary gland. J Mammary Gland Biol Neoplasia.

[45] Chen XJ, Wu S, Yan RM (2019). The role of the hypoxia-Nrp-1 axis in the activation of M2-like tumor-associated macrophages in the tumor microenvironment of cervical cancer. Mol Carcinog.

[46] Tripathi C, Tewari BN, Kanchan RK (2014). Macrophages are recruited to hypoxic tumor areas and acquire a pro-angiogenic M2-polarized phenotype via hypoxic cancer cell derived cytokines Oncostatin M and Eotaxin. Oncotarget.

[47] Shrivastava R, Asif M, Singh V (2019). M2 polarization of macrophages by Oncostatin M in hypoxic tumor microenvironment is mediated by mTORC2 and promotes tumor growth and metastasis. Cytokine.

[48] Xiao L, He Y, Peng F (2020). Endometrial cancer cells promote M2-like macrophage polarization by delivering exosomal miRNA-21 under hypoxia condition. J Immunol Res.

[49] Wang JX, Choi SYC, Niu X (2020). Lactic acid and an acidic tumor microenvironment suppress anticancer immunity. Int J Mol Sci.

[50] Henze AT, Mazzone M (2016). The impact of hypoxia on tumor-associated macrophages. J Clin Invest.

[51] Chen XJ, Deng YR, Wang ZC (2019). Hypoxia-induced ZEB1 promotes cervical cancer progression via CCL8-dependent tumour-associated macrophage recruitment. Cell Death Dis.

[52] Myers KV, Amend SR, Pienta KJ (2019). Targeting Tyro3, Axl and MerTK (TAM receptors): implications for macrophages in the tumor microenvironment. Mol Cancer.

[53] Jiang C, Cheng Z, Jiang T (2020). MicroRNA-34a inhibits cell invasion and epithelial-mesenchymal transition via targeting AXL/PI3K/AKT/Snail signaling in nasopharyngeal carcinoma. Genes Genom.

[54] Li M, Ye J, Zhao G (2019). Gas6 attenuates lipopolysaccharide-induced TNF-α expression and apoptosis in H9C2 cells through NF-κB and MAPK inhibition via the Axl/PI3K/Akt pathway. Int J Mol Med.

[55] Ma Y, Zhou G, Li M (2018). Long noncoding RNA DANCR mediates cisplatin resistance in glioma cells via activating AXL/PI3K/Akt/NF-κB signaling pathway. Neurochem Int.

[56] Mantovani A, Sozzani S, Locati M (2002). Macrophage polarization: tumor-associated macrophages as a paradigm for polarized M2 mononuclear phagocytes. Trends Immunol.

[57] Yunna C, Mengru H, Lei W, Weidong C. Macrophage M1/M2 polarization. Eur J Pharmacol. 2020; 877:173090 10.1016/j.ejphar.2020.173090 32234529

[58] Ngambenjawong C, Gustafson HH, Pun SH (2017). Progress in tumor-associated macrophage (TAM)-targeted therapeutics. Adv Drug Deliv Rev.

[59] Kovaleva OV, Rashidova MA, Samoilova DV (2021). CHID1 is a novel prognostic marker of non-small cell lung cancer. Int J Mol Sci.

[60] Wu K, Lin K, Li X (2020). Redefining Tumor-associated macrophage subpopulations and functions in the tumor microenvironment. Front Immunol.

[61] Raskov H, Orhan A, Gaggar S, Gögenur I. Cancer-associated fibroblasts and tumor-associated macrophages in cancer and cancer immunotherapy. Front Oncol. 2021; 11:668731 10.3389/fonc.2021.668731 PMC8172975 34094963

[62] Cho H, Seo Y, Loke KM (2018). Cancer-stimulated CAFs enhance monocyte differentiation and protumoral TAM activation via IL6 and GM-CSF secretion. Clin Cancer Res.

[63] Komohara Y, Takeya M (2017). CAFs and TAMs: maestros of the tumour microenvironment. J Pathol.

[64] Gil Z, Billan S (2021). Crosstalk between macrophages and endothelial cells in the tumor microenvironment. Mol Ther.

[65] Zhang S, Xie B, Wang L et al. Macrophage-mediated vascular permeability via VLA4/VCAM1 pathway dictates ascites development in ovarian cancer. J Clin Invest. 2021;131:e140315 10.1172/JCI140315 PMC7843216 33295887

[66] Fang W, Zhou T, Shi H (2021). Progranulin induces immune escape in breast cancer via up-regulating PD-L1 expression on tumor-associated macrophages (TAMs) and promoting CD8(+) T cell exclusion. J Exp Clin Cancer Res.

[67] Yu J, Green MD, Li S (2021). Liver metastasis restrains immunotherapy efficacy via macrophage-mediated T cell elimination. Nat Med.

[68] Zhou J, Li X, Wu X (2018). Exosomes released from tumor-associated macrophages transfer miRNAs that induce a Treg/Th17 cell imbalance in epithelial ovarian cancer. Cancer Immunol Res.

[69] Liu C, Chikina M, Deshpande R (2019). Treg cells promote the SREBP1-dependent metabolic fitness of tumor-promoting macrophages via repression of CD8(+) T cell-derived interferon-γ. Immunity.

[70] Paul S, Chhatar S, Mishra A, Lal G (2019). Natural killer T cell activation increases iNOS(+)CD206(-) M1 macrophage and controls the growth of solid tumor. J Immunother Cancer.

[71] Gabrilovich DI (2017). Myeloid-derived suppressor cells. Cancer Immunol Res.

[72] Loeuillard E, Yang J, Buckarma E (2020). Targeting tumor-associated macrophages and granulocytic myeloid-derived suppressor cells augments PD-1 blockade in cholangiocarcinoma. J Clin Invest.

[73] Biswas S, Mandal G, Roy Chowdhury S (2019). Exosomes produced by mesenchymal stem cells drive differentiation of myeloid cells into immunosuppressive M2-polarized macrophages in breast cancer. J Immunol.

[74] Pritchard A, Tousif S, Wang Y (2020). Lung tumor cell-derived exosomes promote M2 macrophage polarization. Cells.

[75] Jang JH, Kim DH, Lim JM (2020). Breast cancer cell-derived soluble CD44 promotes tumor progression by triggering macrophage IL1β production. Cancer Res.

[76] Cruceriu D, Baldasici O, Balacescu O, Berindan-Neagoe I (2020). The dual role of tumor necrosis factor-alpha (TNF-α) in breast cancer: molecular insights and therapeutic approaches. Cell Oncol (Dordr).

[77] Prueitt RL, Boersma BJ, Howe TM (2007). Inflammation and IGF-I activate the Akt pathway in breast cancer. Int J Cancer.

[78] Zhong Q, Fang Y, Lai Q (2020). CPEB3 inhibits epithelial-mesenchymal transition by disrupting the crosstalk between colorectal cancer cells and tumor-associated macrophages via IL-6R/STAT3 signaling. J Exp Clin Cancer Res.

[79] Dong X, Sun R, Wang J et al. Glutathione S-transferases P1-mediated interleukin-6 in tumor-associated macrophages augments drug-resistance in MCF-7 breast cancer. Biochem Pharmacol. 2020; 182:114289 10.1016/j.bcp.2020.114289 33080187

[80] Eskiler GG, Bezdegumeli E, Ozman Z (2019). IL-6 mediated JAK/STAT3 signaling pathway in cancer patients with cachexia. Bratisl Lek Listy.

[81] Jin S, Mutvei AP, Chivukula IV (2013). Non-canonical Notch signaling activates IL-6/JAK/STAT signaling in breast tumor cells and is controlled by p53 and IKKα/IKKβ. Oncogene.

[82] Ilamathi M, Prabu PC, Ayyappa KA, Sivaramakrishnan V (2016). Artesunate obliterates experimental hepatocellular carcinoma in rats through suppression of IL-6-JAK-STAT signalling. Biomed Pharmacother.

[83] Yao X, Huang J, Zhong H (2014). Targeting interleukin-6 in inflammatory autoimmune diseases and cancers. Pharmacol Ther.

[84] Morgan EL, Macdonald A. Autocrine STAT3 activation in HPV positive cervical cancer through a virus-driven Rac1-NFκB-IL-6 signalling axis. PLoS Pathog. 2019; 15:e1007835 10.1371/journal.ppat.1007835 PMC6608985 31226168

[85] Luan S, An Z, Bi S (2018). Interleukin 6 receptor (IL-6R) was an independent prognostic factor in cervical cancer. Histol Histopathol.

[86] Siersbæk R, Scabia V, Nagarajan S (2020). IL6/STAT3 signaling hijacks estrogen receptor α enhancers to drive breast cancer metastasis. Cancer Cell.

[87] Zhu YM, Woll PJ (2005). Mitogenic effects of interleukin-8/CXCL8 on cancer cells. Future Oncol.

[88] Tong H, Ke JQ, Jiang FZ (2016). Tumor-associated macrophage-derived CXCL8 could induce ERα suppression via HOXB13 in endometrial cancer. Cancer Lett.

[89] Shah N, Jin K, Cruz LA (2013). HOXB13 mediates tamoxifen resistance and invasiveness in human breast cancer by suppressing ERα and inducing IL-6 expression. Cancer Res.

[90] den Boon JA, Pyeon D, Wang SS (2015). Molecular transitions from papillomavirus infection to cervical precancer and cancer: role of stromal estrogen receptor signaling. Proc Natl Acad Sci USA.

[91] Wang Y, Yang J, Gao Y (2005). Regulatory effect of e2, IL-6 and IL-8 on the growth of epithelial ovarian cancer cells. Cell Mol Immunol.

[92] Yang J, Wang Y, Gao Y (2009). Reciprocal regulation of 17beta-estradiol, interleukin-6 and interleukin-8 during growth and progression of epithelial ovarian cancer. Cytokine.

[93] Riabov V, Gudima A, Wang N (2014). Role of tumor associated macrophages in tumor angiogenesis and lymphangiogenesis. Front Physiol.

[94] Tsutsui S, Yasuda K, Suzuki K (2005). Macrophage infiltration and its prognostic implications in breast cancer: the relationship with VEGF expression and microvessel density. Oncol Rep.

[95] Oplawski M, Dziobek K, Zmarzły N (2019). Expression profile of VEGF-C, VEGF-D, and VEGFR-3 in different grades of endometrial cancer. Curr Pharm Biotechnol.

[96] Van Trappen PO, Steele D, Lowe DG (2003). Expression of vascular endothelial growth factor (VEGF)-C and VEGF-D, and their receptor VEGFR-3, during different stages of cervical carcinogenesis. J Pathol.

[97] Leek RD, Talks KL, Pezzella F (2002). Relation of hypoxia-inducible factor-2 alpha (HIF-2 alpha) expression in tumor-infiltrative macrophages to tumor angiogenesis and the oxidative thymidine phosphorylase pathway in Human breast cancer. Cancer Res.

[98] Hu H, Hu J, Yang Y (2021). Assessment of circulating HISLA as a potential biomarker for breast cancer diagnosis and prognosis. Clin Exp Med.

[99] Yin M, Li X, Tan S (2016). Tumor-associated macrophages drive spheroid formation during early transcoelomic metastasis of ovarian cancer. J Clin Invest.

[100] Ishihara D, Dovas A, Hernandez L (2013). Wiskott-Aldrich syndrome protein regulates leukocyte-dependent breast cancer metastasis. Cell Rep.

[101] Jing X, Peng J, Dou Y (2019). Macrophage ERα promoted invasion of endometrial cancer cell by mTOR/KIF5B-mediated epithelial to mesenchymal transition. Immunol Cell Biol.

[102] Gonzalez-Avila G, Sommer B, García-Hernández AA, Ramos C (2020). Matrix metalloproteinases' role in tumor microenvironment. Adv Exp Med Biol.

[103] Azevedo Martins JM, Rabelo-Santos SH, do Amaral Westin MC, Zeferino LC. Tumoral and stromal expression of MMP-2, MMP-9, MMP-14, TIMP-1, TIMP-2, and VEGF-A in cervical cancer patient survival: a competing risk analysis. BMC Cancer. 2020; 20: 660 10.1186/s12885-020-07150-3 PMC7364527 32669083

[104] Pelekanou V, Villarroel-Espindola F, Schalper KA (2018). CD68, CD163, and matrix metalloproteinase 9 (MMP-9) co-localization in breast tumor microenvironment predicts survival differently in ER-positive and -negative cancers. Breast Cancer Res.

[105] Wen Z, Liu H, Li M (2015). Increased metabolites of 5-lipoxygenase from hypoxic ovarian cancer cells promote tumor-associated macrophage infiltration. Oncogene.

[106] Sun XY, Han XM, Zhao XL (2019). MiR-93-5p promotes cervical cancer progression by targeting THBS2/MMPS signal pathway. Eur Rev Med Pharmacol Sci.

[107] Zhu D, Ye M, Zhang W (2015). E6/E7 oncoproteins of high risk HPV-16 upregulate MT1-MMP, MMP-2 and MMP-9 and promote the migration of cervical cancer cells. Int J Clin Exp Pathol.

[108] Hildenbrand R, Jansen C, Wolf G (1998). Transforming growth factor-beta stimulates urokinase expression in tumor-associated macrophages of the breast. Lab Invest.

[109] Mahmood N, Mihalcioiu C, Rabbani SA (2018). Multifaceted role of the urokinase-type plasminogen activator (uPA) and its receptor (uPAR): diagnostic, prognostic, and therapeutic applications. Front Oncol.

[110] Hildenbrand R, Wolf G, Böhme B (1999). Urokinase plasminogen activator receptor (CD87) expression of tumor-associated macrophages in ductal carcinoma in situ, breast cancer, and resident macrophages of normal breast tissue. J Leukoc Biol.

[111] Hao Y, Baker D, Ten Dijke P (2019). TGF-β-mediated epithelial-mesenchymal transition and cancer metastasis. Int J Mol Sci.

[112] Steitz AM, Steffes A, Finkernagel F (2020). Tumor-associated macrophages promote ovarian cancer cell migration by secreting transforming growth factor beta induced (TGFBI) and tenascin C. Cell Death Dis.

[113] Li S, Ma YM, Zheng PS, Zhang P (2018). GDF15 promotes the proliferation of cervical cancer cells by phosphorylating AKT1 and Erk1/2 through the receptor ErbB2. J Exp Clin Cancer Res.

[114] Mannino MH, Zhu Z, Xiao H (2015). The paradoxical role of IL-10 in immunity and cancer. Cancer Lett.

[115] Saraiva M, O'Garra A (2010). The regulation of IL-10 production by immune cells. Nat Rev Immunol.

[116] Liu Q, Yang C, Wang S (2020). Wnt5a-induced M2 polarization of tumor-associated macrophages via IL-10 promotes colorectal cancer progression. Cell Commun Signal.

[117] Berti FCB, Pereira APL, Cebinelli GCM (2017). The role of interleukin 10 in human papilloma virus infection and progression to cervical carcinoma. Cytokine Growth Factor Rev.

[118] Torres-Poveda K, Bahena-Román M, Madrid-González C (2014). Role of IL-10 and TGF-β1 in local immunosuppression in HPV-associated cervical neoplasia. World J Clin Oncol.

[119] Lepique AP, Daghastanli KR, Cuccovia IM, Villa LL (2009). HPV16 tumor associated macrophages suppress antitumor T cell responses. Clin Cancer Res.

[120] Oft M (2014). IL-10: master switch from tumor-promoting inflammation to antitumor immunity. Cancer Immunol Res.

[121] Ruffell B, Chang-Strachan D, Chan V (2014). Macrophage IL-10 blocks CD8+ T cell-dependent responses to chemotherapy by suppressing IL-12 expression in intratumoral dendritic cells. Cancer Cell.

[122] Chu PY, Wang SM, Chen PM (2020). Expression of MTDH and IL-10 is an independent predictor of worse prognosis in ER-negative or PR-negative breast cancer patients. J Clin Med.

[123] Qu QX, Huang Q, Shen Y (2016). The increase of circulating PD-L1-expressing CD68(+) macrophage in ovarian cancer. Tumour Biol.

[124] Rowswell-Turner RB, Singh RK, Urh A (2021). HE4 overexpression by ovarian cancer promotes a suppressive tumor immune microenvironment and enhanced tumor and macrophage PD-L1 expression. J Immunol.

[125] Qu QX, Xie F, Huang Q, Zhang XG (2017). Membranous and cytoplasmic expression of PD-L1 in ovarian cancer cells. Cell Physiol Biochem.

[126] Zhao R, Song Y, Wang Y et al. PD-1/PD-L1 blockade rescue exhausted CD8+ T cells in gastrointestinal stromal tumours via the PI3K/Akt/mTOR signalling pathway. Cell Prolif. 2019; 52: e12571 10.1111/cpr.12571 PMC6536456 30714229

[127] Zhu X, Shen H, Yin X (2019). Macrophages derived exosomes deliver miR-223 to epithelial ovarian cancer cells to elicit a chemoresistant phenotype. J Exp Clin Cancer Res.

[128] Mandic A, Usaj Knezevic S, Kapicl IT (2014). Tissue expression of VEGF in cervical intraepithelial neoplasia and cervical cancer. J Buon.

[129] Rahma OE, Hodi FS (2019). The intersection between tumor angiogenesis and immune suppression. Clin Cancer Res.

[130] McCloskey E, Paterson AH, Powles T, Kanis JA. Clodronate. Bone. 2021; 143: 115715. 10.1016/j.bone.2020.115715 33127577

[131] Wang C, La L, Feng H (2020). Aldose reductase inhibitor engeletin suppresses pelvic inflammatory disease by blocking the phospholipase C/protein kinase C-dependent/NF-κB and MAPK cascades. J Agric Food Chem.

[132] Wang H, Jiang Z, Pang Z (2021). Engeletin protects against TNF-α-induced apoptosis and reactive oxygen species generation in chondrocytes and alleviates osteoarthritis in vivo. J Inflamm Res.

[133] Bai H, Yin H (2020). Engeletin suppresses cervical carcinogenesis in vitro and in vivo by reducing NF-κB-dependent signaling. Biochem Biophys Res Commun.

[134] Cannarile MA, Weisser M, Jacob W (2017). Colony-stimulating factor 1 receptor (CSF1R) inhibitors in cancer therapy. J Immunother Cancer.

[135] Puar N, Chovatiya R, Paller AS (2021). New treatments in atopic dermatitis. Ann Allergy Asthma Immunol.

[136] Alotaibi MR, Hassan ZK, Al-Rejaie SS (2018). Characterization of apoptosis in a breast cancer cell line after IL-10 silencing. Asian Pac J Cancer Prev.

[137] Matsunaga MC, Yamauchi PS (2016). IL-4 and IL-13 inhibition in atopic dermatitis. J Drugs Dermatol.

[138] Tovar JM, Bazaldua OV, Vargas L, Reile E (2008). Human papillomavirus, cervical cancer, and the vaccines. Postgrad Med.

[139] Che Y, Yang Y, Suo J (2020). Induction of systemic immune responses and reversion of immunosuppression in the tumor microenvironment by a therapeutic vaccine for cervical cancer. Cancer Immunol Immunother.

[140] Petrillo M, Zannoni GF, Martinelli E et al. Polarisation of tumor-associated macrophages toward M2 phenotype correlates with poor response to chemoradiation and reduced survival in patients with locally advanced cervical cancer. PLoS ONE. 2015; 10: e0136654. 10.1371/journal.pone.0136654 PMC4559430 26335330

[141] Jayasingam SD, Citartan M, Thang TH (2019). Evaluating the polarization of tumor-associated macrophages into M1 and M2 phenotypes in human cancer tissue: technicalities and challenges in routine clinical practice. Front Oncol.

[142] Cheong JE, Sun L (2018). Targeting the IDO1/TDO2-KYN-AhR pathway for cancer immunotherapy—challenges and opportunities. Trends Pharmacol Sci.

[143] Lou Q, Liu R, Yang X (2019). miR-448 targets IDO1 and regulates CD8(+) T cell response in human colon cancer. J Immunother Cancer.

[144] Kaul NC, Mohapatra SR, Adam I (2020). Hypoxia decreases the T helper cell-suppressive capacity of synovial fibroblasts by downregulating IDO1-mediated tryptophan metabolism. Rheumatology (Oxford).

[145] Toulmonde M, Penel N, Adam J (2018). Use of PD-1 targeting, macrophage infiltration, and IDO pathway activation in sarcomas: a phase 2 clinical trial. JAMA Oncol.

[146] Yang SL, Tan HX, Niu TT (2021). The IFN-γ-IDO1-kynureine pathway-induced autophagy in cervical cancer cell promotes phagocytosis of macrophage. Int J Biol Sci.

[147] Pallotta MT, Rossini S, Suvieri C (2021). Indoleamine 2,3-dioxygenase 1 (IDO1): an up-to-date overview of an eclectic immunoregulatory enzyme. FEBS J.

[148] Feng X, Tang R, Zhang R (2020). A comprehensive analysis of IDO1 expression with tumour-infiltrating immune cells and mutation burden in gynaecologic and breast cancers. J Cell Mol Med.

[149] Huang Q, Xia J, Wang L (2018). miR-153 suppresses IDO1 expression and enhances CAR T cell immunotherapy. J Hematol Oncol.

[150] Jiao R, Zheng X, Sun Y (2020). IDO1 expression increased after neoadjuvant therapy predicts poor pathologic response and prognosis in esophageal squamous cell carcinoma. Front Oncol.

[151] Morrissey MA, Kern N, Vale RD (2020). CD47 ligation repositions the inhibitory receptor SIRPA to suppress integrin activation and phagocytosis. Immunity.

[152] Weiskopf K (2017). Cancer immunotherapy targeting the CD47/SIRPα axis. Eur J Cancer.

[153] Liu Y, Chang Y, He X (2020). CD47 enhances cell viability and migration ability but inhibits apoptosis in endometrial carcinoma cells via the PI3K/Akt/mTOR Signaling pathway. Front Oncol.

[154] Shimizu A, Sawada K, Kobayashi M (2021). Exosomal CD47 plays an essential role in immune evasion in ovarian cancer. Mol Cancer Res.

[155] Willingham SB, Volkmer JP, Gentles AJ (2012). The CD47-signal regulatory protein alpha (SIRPa) interaction is a therapeutic target for human solid tumors. Proc Natl Acad Sci USA.

[156] Huang Y, Lv SQ, Liu PY (2020). A SIRPα-Fc fusion protein enhances the antitumor effect of oncolytic adenovirus against ovarian cancer. Mol Oncol.

[157] Samanta D, Park Y, Ni X (2018). Chemotherapy induces enrichment of CD47(+)/CD73(+)/PDL1(+) immune evasive triple-negative breast cancer cells. Proc Natl Acad Sci USA.

[158] Candas-Green D, Xie B, Huang J (2020). Dual blockade of CD47 and HER2 eliminates radio-resistant breast cancer cells. Nat Commun.

[159] Kaur S, Elkahloun AG, Singh SP (2016). A function-blocking CD47 antibody suppresses stem cell and EGF signaling in triple-negative breast cancer. Oncotarget.

[160] Chen M, Miao Y, Qian K (2021). Detachable liposomes combined immunochemotherapy for enhanced triple-negative breast cancer treatment through reprogramming of tumor-associated macrophages. Nano Lett.

[161] Hubert P, Roncarati P, Demoulin S et al. Extracellular HMGB1 blockade inhibits tumor growth through profoundly remodeling immune microenvironment and enhances checkpoint inhibitor-based immunotherapy. J Immunother Cancer. 2021; 9:e001966. 10.1136/jitc-2020-001966 PMC7959241 33712445

[162] Wang X, Gao S, Song L (2021). Astragaloside IV antagonizes M2 phenotype macrophage polarization-evoked ovarian cancer cell malignant progression by suppressing the HMGB1-TLR4 axis. Mol Immunol.

[163] Kawai T, Akira S (2010). The role of pattern-recognition receptors in innate immunity: update on Toll-like receptors. Nat Immunol.

[164] Peng J, Tsang JY, Li D (2013). Inhibition of TGF-β signaling in combination with TLR7 ligation re-programs a tumoricidal phenotype in tumor-associated macrophages. Cancer Lett.

[165] Travers M, Brown SM, Dunworth M (2019). DFMO and 5-azacytidine increase M1 macrophages in the tumor microenvironment of murine ovarian cancer. Cancer Res.

[166] Zhao Y, Liu X, Huo M et al. Cetuximab enhances the anti-tumor function of macrophages in an IL-6 dependent manner. Life Sci. 2021; 267: 118953. 10.1016/j.lfs.2020.118953 33359746

[167] Wu G, Ma Z, Cheng Y (2018). Targeting Gas6/TAM in cancer cells and tumor microenvironment. Mol Cancer.

[168] Sun WS, Fujimoto J, Tamaya T (2003). Coexpression of growth arrest-specific gene 6 and receptor tyrosine kinases Axl and Sky in human uterine endometrial cancers. Ann Oncol.

[169] Goyette MA, Duhamel S, Aubert L (2018). The receptor tyrosine kinase AXL is required at multiple steps of the metastatic cascade during HER2-positive breast cancer progression. Cell Rep.

[170] Kasikara C, Davra V, Calianese D (2019). Pan-TAM tyrosine kinase inhibitor BMS-777607 enhances anti-PD-1 mAb efficacy in a murine model of triple-negative breast cancer. Cancer Res.

[171] Falcone I, Conciatori F, Bazzichetto C (2020). AXL receptor in breast cancer: molecular involvement and therapeutic limitations. Int J Mol Sci.

[172] Bonifacio L, Dodds M, Prohaska D (2020). Target-mediated drug disposition pharmacokinetic/pharmacodynamic model-informed dose selection for the first-in-human study of AVB-S6-500. Clin Transl Sci.

[173] Zhao Y, Yu Z, Ma R (2021). lncRNA-Xist/miR-101-3p/KLF6/C/EBPα axis promotes TAM polarization to regulate cancer cell proliferation and migration. Mol Ther Nucl Acids.

[174] Liu X, Meng L, Chen L (2020). IL-6 expression promoted by Poly(I:C) in cervical cancer cells regulates cytokine expression and recruitment of macrophages. J Cell Mol Med.

[175] Lai YS, Wahyuningtyas R, Aui SP, Chang KT (2019). Autocrine VEGF signalling on M2 macrophages regulates PD-L1 expression for immunomodulation of T cells. J Cell Mol Med.

[176] Guan C, Xiao Y, Li K (2019). MMP-12 regulates proliferation of mouse macrophages via the ERK/P38 MAPK pathways during inflammation. Exp Cell Res.

[177] Gona K, Toczek J, Ye Y (2020). Hydroxamate-based selective macrophage elastase (MMP-12) inhibitors and radiotracers for molecular imaging. J Med Chem.

[178] Selman M, Cisneros-Lira J, Gaxiola M (2003). Matrix metalloproteinases inhibition attenuates tobacco smoke-induced emphysema in Guinea pigs. Chest.

[179] Churg A, Wang R, Wang X (2007). Effect of an MMP-9/MMP-12 inhibitor on smoke-induced emphysema and airway remodelling in guinea pigs. Thorax.

[180] Xing RH, Mazar A, Henkin J, Rabbani SA (1997). Prevention of breast cancer growth, invasion, and metastasis by antiestrogen tamoxifen alone or in combination with urokinase inhibitor B-428. Cancer Res.

[181] Kampan NC, Xiang SD, McNally OM (2018). Immunotherapeutic interleukin-6 or interleukin-6 receptor blockade in cancer: challenges and opportunities. Curr Med Chem.

[182] Coward J, Kulbe H, Chakravarty P (2011). Interleukin-6 as a therapeutic target in human ovarian cancer. Clin Cancer Res.

[183] Guo YQ, Lu P, Duan ZF, Zhang Z (2010). Effects of siltuximab on the interleukin-6/Stat3 signaling pathway in ovarian cancer. Zhonghua Fu Chan Ke Za Zhi.

[184] Casneuf T, Axel AE, King P (2016). Interleukin-6 is a potential therapeutic target in interleukin-6 dependent, estrogen receptor-α-positive breast cancer. Breast Cancer (Dove Med Press).

[185] Ge J, Han T, Shan L (2020). Long non-coding RNA THOR promotes ovarian Cancer cells progression via IL-6/STAT3 pathway. J Ovarian Res.

[186] Masjedi A, Hashemi V, Hojjat-Farsangi M (2018). The significant role of interleukin-6 and its signaling pathway in the immunopathogenesis and treatment of breast cancer. Biomed Pharmacother.

[187] Dijkgraaf EM, Heusinkveld M, Tummers B (2013). Chemotherapy alters monocyte differentiation to favor generation of cancer-supporting M2 macrophages in the tumor microenvironment. Cancer Res.

[188] Dijkgraaf EM, Santegoets SJ, Reyners AK (2015). A phase I trial combining carboplatin/doxorubicin with tocilizumab, an anti-IL-6R monoclonal antibody, and interferon-α2b in patients with recurrent epithelial ovarian cancer. Ann Oncol.

[189] Huynh LK, Hipolito CJ, Ten Dijke P (2019). A perspective on the development of TGF-β inhibitors for cancer treatment. Biomolecules.

[190] Colak S, Ten Dijke P (2017). Targeting TGF-β signaling in cancer. Trends Cancer.

[191] Llopiz D, Ruiz M, Silva L (2021). Inhibition of adjuvant-induced TAM receptors potentiates cancer vaccine immunogenicity and therapeutic efficacy. Cancer Lett.

[192] Klichinsky M, Ruella M, Shestova O (2020). Human chimeric antigen receptor macrophages for cancer immunotherapy. Nat Biotechnol.

[193] Zhang L, Tian L, Dai X (2020). Pluripotent stem cell-derived CAR-macrophage cells with antigen-dependent anti-cancer cell functions. J Hematol Oncol.

[194] Wang D, Xue M, Chen J et al. Macrophage-derived implantable vaccine prevents postsurgical tumor recurrence. Biomaterials. 2021; 278: 121161. 10.1016/j.biomaterials.2021.121161 34601198

[195] Gu Z, Liu T, Liu C (2021). Ferroptosis-strengthened metabolic and inflammatory regulation of tumor-associated macrophages provokes potent tumoricidal activities. Nano Lett.

[196] Li J, Jiang X, Li H et al. Tailoring materials for modulation of macrophage fate. Adv Mater 2021; 33: e2004172. 10.1002/adma.202004172 PMC9245340 33565154

[197] Kang M, Lee SH, Kwon M et al. Nanocomplex-mediated in vivo programming to chimeric antigen receptor-M1 macrophages for cancer therapy. Adv Mater. 2021; e2103258. 10.1002/adma.202103258 34510559

[198] Chen C, Song M, Du Y (2021). Tumor-associated-macrophage-membrane-coated nanoparticles for improved photodynamic immunotherapy. Nano Lett.

[199] Wang B, Zhang W, Zhou X (2019). Development of dual-targeted nano-dandelion based on an oligomeric hyaluronic acid polymer targeting tumor-associated macrophages for combination therapy of non-small cell lung cancer. Drug Deliv.

[200] Wei B, Pan J, Yuan R (2021). Polarization of tumor-associated macrophages by nanoparticle-loaded *Escherichia coli* combined with immunogenic cell death for cancer immunotherapy. Nano Lett.

